# Developing lactic acid bacteria starter cultures for wholemeal rye flour bread with improved functionality, nutritional value, taste, appearance and safety

**DOI:** 10.1371/journal.pone.0261677

**Published:** 2022-01-14

**Authors:** Dorota Litwinek, Jakub Boreczek, Halina Gambuś, Krzysztof Buksa, Wiktor Berski, Magdalena Kowalczyk

**Affiliations:** 1 Department of Carbohydrate Technology and Cereal Processing, Faculty of Food Technology, University of Agriculture in Krakow, Krakow, Poland; 2 Institute of Biochemistry and Biophysics, Polish Academy of Sciences, Warsaw, Poland; Universidad Autonoma de Chihuahua, MEXICO

## Abstract

Starter cultures composed of lactic acid bacteria (LAB) were developed based on the genotypic and phenotypic characterisation of isolates belonging to dominant groups of bacteria in spontaneous rye wholemeal sourdoughs. Combinations of strains have been evaluated on an industrial scale in the sourdough fermentation process. Wholemeal rye bread was prepared using sourdoughs obtained with 3 new starter cultures, and compared to bread made using the commercial culture (LV2). All newly developed cultures used for the preparation of wholemeal rye bread allowed to obtain better quality products as compared to the LV2 based bread. The best results were obtained when the culture containing *Lactiplantibacillus plantarum* 2MI8 and exopolysaccharide (EPS)-producing *Weissella confusa*/*cibaria* 6PI3 strains was applied. The addition of yeast during sourdough breads production, especially the one prepared from mentioned above starter culture, significantly improved their organoleptic properties, their volume and crumb moisture was increased, and also the crumb acidity and hardness was reduced. Fermentation of rye wholemeal dough, especially without the yeast addition, resulted in a significant reduction in the content of higher inositol phosphates as compared to the applied flour, which is associated with improved bioavailability of minerals. The results of this study prove that the investigated new starter cultures can be successfully applied in wholemeal rye bread production.

## Introduction

Sourdoughs used in bakery industry can be spontaneous [1] or commercial ones, such as San Francisco acid used in wheat bread production [2], or Böcker–Reinzucht–Sauer (BRS) acid for rye bread [3]. Lately, starter cultures become increasingly popular in bread manufacturing [4–6]. They are mainly composed of bacteria from commercial culture collections, or bacterial strains isolated in an appropriate manner from bakery sourdough, i.e. from the same environment in which they will be later applied. The microbial biodiversity in sourdough used for bacteria isolation depends on many factors e.g. autochthonous microbiota, type and composition of flour, and also on the method of its preparation. It is important that the flour used to prepare the “mother” sourdough (from which the microorganisms are isolated) has similar properties (i.e. degree of grinding) to the one, that is to be used for the bread production [4, 7]. Appropriate sourdough preparation, as well as, careful selection of strains isolated for the purpose of starter culture development guarantee preparation of a desire mixture of microorganisms, that will enable to start a proper fermentation process in the same environment, e.g. in a new sourdough, and to obtain bread with a proper quality features [4, 5].

Recently, it is observed a growing demand to provide a food possessing health promoting properties. Rye bread prepared on sourdough can be treated as such product, especially when wholegrain flour was used for baking. Rye is a cereal with long tradition of use in technology and nutrition in Northern, Central and Eastern Europe [8]. Rye endosperm and flours contain relatively less starch and proteins, but more fibre than wheat with proven health benefits [9]. The Commission Regulation (EU) No. 432/2012 [10] lays down the admissible health claims and conditions of use of the claims specifically for rye fibre: ’Rye fibre contributes to normal bowel function’. The intake of wholegrain cereal products, including rye ones, is associated with a reduced risk of development of many civilization diseases and health disorders: a reduction in the risk of type 2 diabetes in adults, induction of the feeling of satiety and a decrease in the inclination to overeat [8, 11]. Rye bread should be prepared with the use of sourdough, due to necessity of pentosans decomposition, and additional functional features can be obtained when sourdough is applied [12]. Despite nutritional benefits resulting from rye products intake, their consumption is decreasing, therefore, it is essential to constantly accentuate advantages of the consumption of rye, rye bread and baked products and to support them by scientific arguments.

Application of sourdough in bread manufacturing results in hydrolysis of dietary fibre, reduced lipid rancidity, and enables an increase in protein and starch digestibility, and also an increase in vitamin levels and mineral bioavailability is observed [13]. Phytic acid, also called inositol hexakisphosphate (IP6), is present in cereals in the form of complexes with metal cations and proteins. Its enzymatic degradation requires an optimum pH which can be provided by natural fermentation. Such a degradation of IP6 can significantly increase the amount of soluble iron, zinc and calcium in fermented cereal products [14].

Sourdough application reduces possible pathogenic bacterial contamination in bakery products, mostly due to pH lowering [15, 16], and also LAB demonstrate the ability of mycotoxins decomposition [17]. Moreover, bioactive compounds are synthesized during fermentation lactic acid (LA), while ingredients that hinder the digestion of grain-based products or digestion-related pathologies, such as gluten sensitivity or gastrointestinal syndromes, are diminished [13]. Although the impact of sourdough bread consumption on human health has not been fully studied, Abbondio et al. [18] provide evidence that consumption of sourdough-leavened bread has the potential to significantly change the taxonomy of the gut microbiota and the metabolic functions of some of its most important members, including *Bacteroides* and *Clostridium* [13].

In stable sourdough ecosystems, yeast, and homofermentative and heterofermentative lactobacilli representing lactic acid bacteria (LAB) occur, but heterofermentative LAB are the most important, because except LA they also produce acetic and succinic acids, ethanol, acetoin, diacetyl, acetone as well as ethylene glycol, carbon monoxide and hydrogen. All mentioned above compounds contribute to the proper dough structure, regulate enzymatic activity and determine the proper taste and smell of the bread [19]. The vast majority of microorganism are lactobacilli, in a much smaller amount there are other microbes of the genera: *Lactococcus*, *Pedicoccu*s, *Weissella*, *Carnobacterium* and *Leuconosto*c [4, 20–22].

In order to determine the number of microorganisms and their growth dynamics during the sourdough development, a thorough examination of microorganisms, i.e. bacteria and yeast, was performed in the environment of spontaneous rye sourdough prepared from rye, wheat and spelt white flours [23, 24] and wholemeal flour [25]. However these authors did not perform baking tests using isolated bacteria, either individually or as a mixture, and did not evaluate their influence on bread crumb texture. In previous work [7] microbial diversity and the community growth dynamics in rye sourdoughs made from wholegrain flour were evaluated using culture-dependent and culture-independent approaches.

The aim of the present study was to select bacterial strains isolated by Boreczek et al. [7] from sourdoughs made from wholemeal rye flour for new starter cultures and to examine their effect during industrial production of the wholemeal rye bread. The developed starter cultures could complement the insufficient supply of the bakery starter cultures currently present on the market. The resulting bread is expected to possess high sensory and textural quality on the day of baking and during storage, and increased nutritional value.

## Materials

A total of 57 LAB isolates (lactobacilli—30, *Pediococcus pentosaceus* -11, *Weissella* sp.—7, *Leuconostoc* sp.—3, *Lactococcus lactis*—6) and spore-forming, bread spoilage bacterial strains belonging to *Bacillus cereus*, *Brevibacillus brevis* or *Lysinibacillus fusiformis*, used in this study had been isolated previously in our group from spontaneous wholemeal sourdoughs produced in the Polish local bakery. Unique strains were deposited in the Central Collection of Strains (COLIBB) of the Institute of Biochemistry and Biophysics, Polish Academy of Sciences (IBB PAS) in Warsaw. The material for sourdoughs and bread making was wholemeal rye flour of the following properties determined as described by Litwinek et al. [26]: falling number– 204 s, fat acidity—36 mg KOH/100 g db, the content of: total protein—7.94%; crude lipid– 2.11%; ash 1.58%; dietary fibre 16.84%; total myo-inositol phosphates—1.18% db, including inositol hexakisphosphate (IP_6_)—0.87% db. Sourdoughs were prepared using starter cultures (Table 1) developed under this study which were marked with the symbols: XI, XII, XIIB and commercial starter dedicated to rye flour souring [27]—LV2 (Lesaffre, France).

**Table 1:** Starter cultures’ composition.

Name	Composition	Strain no. in IBB PAS collection	Strain no. in PCM* collection	Percentage of bacteria in culture
**C** **	***Levilactobacillus brevis***	-	-	-
	***Saccharomyces chevalieri***	-	-	-
**XI**	***Lactiplantibacillus plantarum* 2MI8**	IBB3249	B/00117	33%
	***Lactiplantibacillus plantarum* 6PIII6B**	IBB3255	B/00118	33%
	***Levilactobacillus brevis* 2MIII4**	IBB3227	B/00119	33%
**XII**	***Lactiplantibacillus plantarum* 2MI8**	IBB3249	B/00117	25%
	***Lactiplantibacillus plantarum* 6PIII6B**	IBB3255	B/00118	25%
	***Levilactobacillus brevis* 2MIII4**	IBB3227	B/00119	25%
	***Weissella confusa*/*cibaria* 6PI3**	IBB3282	B/00120	25%
**XIIB**	***Lactiplantibacillus plantarum* 2MI8**	IBB3255	B/00117	75%
	***Weissella confusa*/*cibaria* 6PI3**	IBB3282	B/00120	25%

*Polish Collection of Microorganisms (WFCC, No. 106).

**Commercial starter culture—LV2 from Lesaffre [27].

## Methods

### Standard growth conditions of bacterial strains

Lactococci, other LAB and spore-forming bacterial strains were maintained as frozen stocks at -80°C in M17 broth (Oxoid, Basingstoke, UK) supplemented with 0.5% (w/v) glucose (GM17), de Man, Rogosa, and Sharpe (MRS) broth (Merck, Darmstadt, Germany) or Luria-Bertani (LB) broth (Difco Laboratories, Franklin Lakes, New Jersey, USA), respectively; in the presence of 15% (v/v) glycerol as cryoprotective agent. Purity was checked by plating strains on corresponding agar media and microscopic examination. LAB working cultures were started from single colonies and cultivated overnight (o/n) in GM17 liquid medium at 30°C for *Lactococcus*, in MRS broth at 30°C for *Weissella* and *Pediococcus* or at 37°C for lactobacilli. Lactobacilli were grown under anaerobic conditions in 2.5-liter jars containing GENbox anaer sachets (bioMérieux, Marcy l’Etoile, France). Spore-forming bacteria were grown o/n in LB broth at 30°C.

### DNA extraction from bacteria

The volume of 2 ml of each LAB strain working cultures (approximately 10^7^ CFU) were pelleted by centrifugation at 12 500 × g for 5 min. The pellets were washed with TES buffer pH 8.0 (25 mM Tris, 10 mM EDTA, 50 mM sucrose). The enzymatic lysis was performed as lysis C described by Kowalczyk et al. [28]: washed cell pellets were resuspended in 300 μl of TES buffer with lysozyme (20 mg ml^-1^; Sigma-Aldrich, Saint Louis, Missouri, USA) and 15 μl of mutanolysin (stock 1000 U ml^-1^; Sigma-Aldrich, Saint Louis, Missouri, USA). Tubes were incubated at 37°C for 60 min. Next steps were carried out by using a GenomicMini kit (A&A Biotechnology, Poland) in accordance with the manufacturer’s instructions. Obtained DNA was diluted with 30 μl sterile distilled water and stored at -20°C until use. The quality of the DNA extracted was checked by agarose gel electrophoresis. The concentration and purity of DNA were assessed using Spectrophotometer NanoDrop ND-1000 (NanoDrop Technologies, Wilmington, Delaware, USA).

### Randomly amplified polymorphic DNA (RAPD)-PCR

Two oligonucleotides, B10 (5’-CTGCTGCTGGGAC-3’) [29, 30] and GACA (5’-GACAGACAGACAGACA-3’) [31, 32] microsatellite primers were used for isolates biotyping. Amplification reactions were performed in a 30 μl reaction volume containing: 3 μl of 10x ExTaq buffer, 250 μmol l^-1^ each of dATP, dCTP, dGTP and dTTP, 1 μmol l^-1^ primer, 100 ng genomic DNA and 2 U ExTaq DNA polymerase (TaKaRa Bio inc, Kusatsu, Japan). Amplification was performed in a C1000^™^ Thermal Cycler (Bio-Rad, Hercules, California, USA) as follows: initial denaturation at 94°C for 5 min, followed by 29 cycles of denaturation at 94°C for 1 min, primer annealing at 42°C for 1 min, and extension of the primer at 72°C for 2 min. Finally, the mixture was held at 72°C for 7 min to allow complete extension of amplified products. Then, 15 μl of PCR products were separated by electrophoresis on 0.8% (w/v) agarose (BioShop, Burlington, Ontario, Canada) gel for 3 hr (60 V) at 20°C containing ethidium bromide (0.1 μg ml^-1^; Merck, Darmstadt, Germany), and the DNA was detected by UV transillumination. GeneRuler 1 kb DNA Ladder (Thermo Scientific, Waltham, Massachusetts, USA) was used as a DNA molecular weight marker. The analysis was performed in two repeats for each primer.

### RAPD-PCR data analysis

The images of the gels were captured using the G-BOX (Syngene, Cambridge, UK). The images were then saved as TIFF files and exported into the pattern GelJ analysis software (version 2.0) for processing. Calculation of similarity of the PCR fingerprinting profiles were based on the DICE Pearson product-moment correlation coefficient. Dendrograms were deduced from the matrix of similarities by the unweighted pair group method using arithmetic average (UPGMA) clustering algorithm [33].

### API tests

Carbohydrate fermentation reactions were recorded using API 50 CHL galleries (BioMe´rieux, Marcy l’Etoile, France), according to the manufacturer’s instructions. Readings (changes in colour from violet) were monitored after 24 and 48 hr at 30°C. Positive results were marked as ˮ+ˮ or ˮ+/-ˮ, where ˮ+ˮ denoted a complete change to yellow and ˮ+/-ˮ partial change (closer to yellow, than to violet). Esculin hydrolysis (revealed by a change to a darker colour or black) was represented by a positive sign (+).

### Screening for the production of exopolysaccharides (EPS)

The ability for production of EPS was evaluated on M17 (for *L*. *lactis*) or MRS without dextrose (Condalab, Madrid, Spain) agar media, both supplemented with 10% sucrose after 72-h incubation at 30°C. The strains which produced slimy colonies were regarded as being EPS positive [34].

### Coexistence tests

The antagonistic activity of tested strains against spore-forming, bread spoilage bacteria or other LAB strains selected for starter cultures were examined by an inhibitory spectrum assay. To this end, indicator, spore-forming bacteria or LAB were inoculated and grown o/n in appropriate liquid medium. Then 60 μl of the o/n culture was used to inoculate 60 ml of cooled corresponding agar medium which was immediately poured onto a 140 mm plate. Following agar setting, 5 μl of tested LAB strain was spotted on selected locations on the plate. The plates were allowed to absorb the drops and were incubated o/n at 30°C. The antagonism against strain was visualized by the observation of an inhibition zone. The presence of inhibition zone was denoted as “++”, ˮ+ˮ or ˮ+/-ˮ, depending on the size, whereas lack of antagonistic activity was denoted as ˮ-ˮ.

### Biomass production of LAB strains selected for starter cultures

The biomass of particular selected strains was produced separately in 2-l bioreactors—BIOSTAT^®^ B plus (Sartorius Stedim Biotech, Goettingen, Germany). Lactobacilli strains were cultured according to conditions described by Xiong et al. [35] with slight modifications by inoculating the MRS medium supplemented with 2% glucose with 3% inoculum of the working culture. The biomass of *Weissella* strain was produced in the MRS medium supplemented with sodium citrate (3 g l^-1^) and Tween 80 (0.5 ml l^-1^) by using 10% inoculum based on Ricciardi et al. [36]. Fermentation processes were carried out at 37°C for lactobacilli and at 35°C for *Weissella*; for lactobacilli, a fed-batch system was used with carbon source supplementation (3 g glucose h^-1^) for 16 hr (from 12 to 28 hr). The multiplication of the bacterial biomass was carried out without oxygenation and aeration, at agitation speed of 100 rpm. The pH was monitored and controlled at 6.5 with 10 M NaOH solution. The process was carried out for 30 hr for lactobacilli and 7 hr for *Weissella*. In order to concentrate the biomass, the output from each fermentor was centrifuged at 4°C, at 6 250 × g for 15 min., then washed with 0.9% NaCl solution and recentrifuged. The obtained pellet was suspended in a solution with cryoprotective properties (0.9% NaCl, 20% trehalose) and frozen in liquid nitrogen. The bacteria aliquots were stored at -80°C. The number of bacteria in the frozen samples was determined by plating 10-fold dilutions in 0.9% NaCl on a solid medium and determining viable cell counts (CFU ml^-1^). In order to obtain starter cultures, the selected frozen samples were thawed in water bath at 30°C and the biomass of bacterial strains was mixed in appropriate proportions. The resulting sets of strains were re-frozen in liquid nitrogen and stored at -80°C. The number of bacteria was confirmed by the already described method shortly before use and the starter cultures were transported on dry ice and stored in the bakery at -22°C until use.

### Sourdough preparation

The above mentioned starter cultures were used to prepare the starters from wholemeal rye flour and then the sourdoughs. To prepare 100 kg of sourdough in a BIOFM-Ż 400 type industrial fermentor (BioStar PPHU, Poland), the following ingredients were used: 40 kg of flour (including 4 kg in the starter) and 60 kg of water (including 6 kg in the starter) and the commercial starter culture LV2–20 g (2 × 10^9^ CFU of bacteria and 2 × 10^9^ CFU of yeast) or 1 ml of the starter culture (10^10^ CFU of bacteria). The obtained sourdoughs were designated by the following symbols: SC, SXI, SXII and SXIIB, according to the starter culture used, respectively: C (LV2), XI, XII and XIIB.

The starter was fermented for 24 hr at about 37°C, and then added to the remaining amount of flour and water placed in sourdough fermentor. It was fermented for another 24 hr at 30°C. During the sourdough fermentation, the acidification process was monitored using a computerized acidifying activity evaluation system (iCINAC, AMS Alliance, Italy), measuring active acidity (pH) every 10 min. In addition, measurements of potential acidity as total titratable acidity (TTA) of sourdough with 0.1 N NaOH expressed in terms Acidity degree as millilitre of NaOH per 100 g, after 24 and 48 hr of fermentation were performed according to the previously described method [26].

### Dough preparation and bread baking

Bread dough prepared with sourdough addition only (BSC, BSXI, BSXII, BSXIIB) was made from 51 kg of wholemeal rye flour, 17 kg of sourdough with the appropriate starter, 1 kg of salt and 31 kg of water. For the dough preparation with sourdough and the yeast addition (breads codified as BSCY, BSXIY, BSXIIY, BSXIIBY) the following ingredients were used: 50 kg of wholemeal rye flour, 17 kg of sourdough, 1 kg of salt, 1 kg of yeast and 31 kg of water. The dough was prepared by mixing the ingredients in a spiral mixer bowl. The resulting dough was manually divided into 0.6 kg dough pieces and formed. The fermentation time for dough pieces obtained solely on sourdough was 240 min, while those obtained with sourdough and yeast addition was 90 min. Bread baking was carried out in an electric baking oven at 200°C for 60 min.

### Bread analysis

The obtained bread was subjected to organoleptic assessment according to PN-A-74108:1996 [37]. The following features were assessed by trained 15-person panel with checked sensory sensitivity (and possible scores were awarded): appearance (5, 4, 0, -35), thickness (3, 2, 0, -35) and color of the crust (4, 3, 0, -35), crust other features (4, 3, 0, -35), elasticity (4, 3, 0, -35), porosity (3, 2, 0, -35), color and cutting ability (3, 2, 0, -35), taste and aroma (6, 5, 0, -35). Basing on the sum of obtained points bread was classified into an appropriate quality class (first quality class was assigned to bread reaching 36 point of 40). The weight of cold loaves was determined and the baking loss was calculated as the difference between the weight of the formed dough piece and the weight of the cooled bread, expressed as a percentage of the dough weight. Bread volume was measured by a three-dimensional using a low-frequency, high-precision laser Volscan Profiler (Stable Microsystems, GB). Based on the bread size in the study, a vertical step size of 2 mm and a rotational speed of 0.5 rps were applied. Moisture of the crumb of bread was measured by drying for 1 hr at 130°C, according to AOAC method 925.10 [38].

Texture parameters were measured using a texture analyzer TA.XT Plus (Stable Microsystems, GB) according to the standard program, at the compression rate 5 mm/s. A sample of slice bread crumb, taken from the central of the loaf with a height 30 mm, was pressed to reach 50% heigh by a P/20 aluminum compression plate, in two cycles with a 5 s delay. From the resulting parameters of TPA, hardness of the crumb was used as indicators of textural properties. The calculations were performed using the attached software Texture Exponent (Stable Microsystems, GB) [39].

In order to determine the changes occuring during the bread storage, obtained breads were stored for 5 days, at constant conditions (23–24°C and relative air humidity 64%) and the analysis of crumb moisture and hardness was performed, on the day of baking and on the fifth day of storage. In fresh bread, total titratable acidity (TTA) of the crumb was evaluated (The acids contained in 15 g of sample were extracted for 1 hr in 100 ml of distilled water, and an aliquot of 50 ml filtrate was titrated against 0.1 N NaOH using phenolphthalein as indicator). TTA was expressed in terms of acidity degree as millilitre of NaOH per 100 g [39].

The chemical composition of bread was determined according to the AOAC methods [38], including: dry mass (925.10), total protein (950.36), total dietary fibre (including soluble and insoluble fraction—935.38), raw fat (930.05), total ash (930.05).

Determination of organic acids was done according to Buksa [40]. In short ten grams of milled rye bread sample was immediately homogenized with 20 ml of distilled water for 15 min, further extracted in closed tubes at room temperature for 45 min, and finally centrifuged for 10 min (12 000 × g, at 4°C). To 10 ml of supernatant 0.3 ml of Carrez I and Carrez II solutions were added, samples were vortexed and centrifuged for 10 min (12 000 × g, at 4°C). The supernatant was filtered through a syringe filter (0.22 μm), and 20 μl of the filtrate was subjected to analysis of OA, alcohols and sugars using HPLC system (Knauer) equipped with Eurokat H (Knauer) column and two detectors RI (Knauer) and UV (working at 210 nm) in series. 0.005 mol/l H2SO4 was used as an eluent at a flow rate of 0.6 ml/min. The injection volume of standard and sample solutions was 20 μl and the separation was done at a column temperature of 60°C. The data acquisition and calculations were made using the software programs Eurochrom (Knauer) and Clarity (DataApex).

Extraction of inositol phosphates from samples was conducted according to Sandberg et al. [41]. Briefly 0.5 g of samples were extracted in 20 ml of 0.5 M hydrochloric acid at 20°C for 3 hr. Extracts were centrifuged for 10 min at 6 000 × g, and separated by ion exchange chromatography using 2 g of AG 1 × X8 anion-exchanger (Bio-Rad). Eluates were evaporated, redissolved in deionized water and injected into HPLC/RI system (Knauer, Germany).

The separation was performed according to Chen and Li [42] by a linear gradient elution program on a CarboPac PA-100 column (250 mm × 4 mm), and the detection by a UV detector at 295 nm after post-column reaction with a solution of 1 g/l Fe(NO3)3 in 0.33 M HClO4. The gradient elution was effected with a mixture of two eluents: (A) 500 mM HCl and (B) H_2_O; 0–16 min, 8–20% A, 92–80% B; 16–33 min, 20–37% A, 80–63% B; 33–49 min, 37–100% A, 63–0% B; 49–50 min, 100% A, 0% B; and 50–50.1 min, 100–8% A, 0–92% B. The flow rates of eluent and post-column reaction solution were 1.0 and 0.4 ml/min, respectively. A knitted coil (200 μl) for post-column reaction was used. The column temperature was maintained at 30°C, and the injection volume of standard and sample solutions was 100 μl. The data acquisition and calculations were made using the software programs Eurochrom (Knauer) and Clarity (DataApex).

### Statistical evaluation of results

All bread analyses were performed at least in duplicate, and the obtained results were subjected to a two-factor analysis of variance (ANOVA) using Statistica 13 statistical software package (StatSoft, TIBCO Software Inc.), differentiating the samples depending on the type of the applied starter culture and the yeast addition. The significance of differences between the average values was verified by Duncan’s test at α ≤ 0.05.

## Results and discussion

### Genotypic and phenotypic characterization of isolates

In order to differentiate LAB strains isolated from spontaneous rye wholemeal sourdoughs [7], the isolates belonging to groups identified by 16S rRNA genes sequencing represented by more than two members were subjected to RAPD-PCR with GACA and B10 primers. The similar RAPD patterns with one to several bands, depending on the species and primer were obtained for both repeats. Similarity dendrograms of the representing RAPD-PCR patterns were analysed by cluster analysis (S1 Fig). For clustering analysis, a cut-off value of 70–80% similarity level is usually adopted [43, 44]. In this work, a cut-off value of 75% as proposed by Venturi et al. [45] was assumed. The B10 primer showed higher discriminatory power than GACA for *Lactiplantibacillus plantarum* [basonym: *Lactobacillus plantarum*] (10/9 clusters), *Levilactobacillus brevis* [basonym: *Lactobacillus brevis*] (7/6 clusters), *P*. *pentosaceus* (6/3 clusters) and for *Weissella* sp. (7/6 clusters). For other groups the same number of clusters were obtained for both primers. The cut-off value of 75% obtained for both used primers was finally used for strain differentiation. Among 14 isolates identified as *L*. *plantarum* two pairs of isolates showed high similarity of patterns with both tested primers (S1A and S1B Fig). In the case of *L*. *brevis* (7 isolates; S1C and S1D Fig), *Companilactobacillus crustorum* [basonym: *Lactobacillus crustorum*] (4 isolates; S1E and S1F Fig) and *Weissella* sp. (7 isolates; S1I and S1J Fig) none of the strains showed similarity at the assumed cut-off value. Among 11 isolates identified as *P*. *pentosaceus* five were classified as the same strain (S1G and S1H Fig). All 3 isolates of *Leuconostoc* sp. showed similarity over 75% (S1K and S1L Fig), whereas 6 isolates of *L*. *lactis* were classified as two strains (S1M and S1N Fig).

Furthermore, all isolates were analysed with API 50 CHL tests. The complete metabolic profiles are shown in S1 Table, whereas the summary of carbohydrate fermentation ability is presented in Table 2. Most of the LAB isolates classified by RAPD-PCR as the same strain, except two *Leuconostoc* sp. and two *L*. *lactis* isolates were differentiated by the phenotypic analysis. Unique strains were deposited in the Central Collection of Strains (COLIBB) at IBB PAS. The combination of RAPD-PCR and API 50 CHL allowed for differentiation of isolates within groups of species and indicated a high biodiversity of LAB at the strain level in rye wholemeal sourdoughs.

**Table 2:** Phenotypic characterization of LAB strains isolated from rye wholemeal sourdoughs.

Groups identified based on sequencing of 16S rRNA genes	IBB PAS culture collection no.	Isolate/Strain	EPS	Antagonistic activity against			No. of metabolized carbon sources		
				*B*. *cereus* 6LII1	*B*. *brevis* 6LIII2	*L*. *fusiformis* 6LI1	+	+/-	Total
*L*. *plantarum*	3247	2MI1	-	+/-	+/-	-	25	0	25
*L*. *plantarum*	3248	2MI3	-	+/-	+/-	-	21	2	23
*L*. *plantarum*	3249	**2MI8**	-	-	-	-	21	0	21
*L*. *plantarum*	3690	2MI9	-	-	+/-	-	24	1	25
*L*. *plantarum*	3691	2MII1	-	+/-	+	-	23	2	25
*L*. *plantarum*	3250	2MII2	-	-	-	-	26	3	29
*L*. *plantarum*	3692	2MII8	-	-	+/-	-	23	2	25
*L*. *plantarum*	3251	2MII11a	-	+/-	++	-	25	1	26
*L*. *plantarum*	3252	2MII12a	-	+/-	++	+/-	24	3	27
*L*. *plantarum*	3693	2MIII2	-	+/-	+	-	23	3	26
*L*. *plantarum*	3253	2MIII3	-	+/-	++	-	20	1	21
*L*. *plantarum*	3254	6PII3B	-	-	+/-	+	19	2	21
*L*. *plantarum*	3694	6PIII1B	-	+/-	+/-	+	21	2	23
*L*. *plantarum*	3255	**6PIII6B**	-	-	+	+	20	0	20
*L*. *brevis*	3224	2MI2a	-	-	-	-	9	0	9
*L*. *brevis*	3225	2MII4	-	-	+	-	13	0	13
*L*. *brevis*	3226	2MII10	-	-	+	-	9	1	10
*L*. *brevis*	3227	**2MIII4**	-	-	++	-	10	1	11
*L*. *brevis*	3228	2MIII8	-	-	++	-	11	1	12
*L*. *brevis*	3229	6PII5	-	-	-	-	10	1	11
*L*. *brevis*	3230	6PIII5	-	-	-	-	10	1	11
*C*. *paralimentarius*	3239	2MIII5b	-	ND	ND	ND	14	0	14
*C*. *paralimentarius*	3240	2MIII6	-	ND	ND	ND	17	2	19
*C*. *crustorum*	3233	2MI4	-	ND	ND	ND	24	1	25
*C*. *crustorum*	3234	2MII6	-	ND	ND	ND	13	1	14
*C*. *crustorum*	3235	2MII9c	-	ND	ND	ND	20	5	25
*C*. *crustorum*	3236	2MIII1	-	ND	ND	ND	22	3	25
*C*. *heilongjiangensis*	3237	2MII9b	-	ND	ND	ND	16	1	17
*C*. *heilongjiangensis*	3238	2MIII5a	-	ND	ND	ND	14	0	14
*L*. *coryniformis*	3231	2MII11c	-	ND	ND	ND	14	0	14
*P*. *pentosaceus*	3267	2MI2c	**-**	ND	ND	ND	16	1	17
*P*. *pentosaceus*	3695	2MI7	**-**	ND	ND	ND	20	0	20
*P*. *pentosaceus*	3268	2MI12	**-**	ND	ND	ND	23	0	23
*P*. *pentosaceus*	3269	2MII7	**-**	ND	ND	ND	17	0	17
*P*. *pentosaceus*	3271	2MIII9	**-**	ND	ND	ND	17	0	17
*P*. *pentosaceus*	3696	2MIII10	**-**	ND	ND	ND	17	0	17
*P*. *pentosaceus*	3272	6PII3A	**-**	ND	ND	ND	17	0	17
*P*. *pentosaceus*	3273	6PII4	**-**	ND	ND	ND	16	0	16
*P*. *pentosaceus*	3274	6PIII1A	**-**	ND	ND	ND	19	0	19
*P*. *pentosaceus*	3697	6PIII6A	**-**	ND	ND	ND	18	0	18
*P*. *pentosaceus*	3275	6PIII7	-	ND	ND	ND	16	2	18
*Weissella* sp.	3281	2EI1	+	+/-	+/-	-	13	3	16
*Weissella* sp.	3282	**6PI3**	+	+/-	+/-	-	13	4	17
*Weissella* sp.	3283	6PI5A	+	-	+/-	-	13	2	15
*Weissella* sp.	3284	6PI5B	+	-	+/-	-	11	5	16
*Weissella* sp.	3285	6PI6A	+	-	+/-	-	9	3	12
*Weissella* sp.	3286	6PI6B	+	+/-	+/-	-	10	4	14
*Weissella* sp.	3287	6PI7	+	+/-	+/-	-	13	4	17
*Leuconostoc* sp.	3263	2EI2	+	ND	ND	ND	17	2	19
*Leuconostoc* sp.	-	2EI3 = 2EI2	+	ND	ND	ND	17	2	19
*Leuconostoc* sp.	3264	2EII1	+	ND	ND	ND	11	4	15
*L*. *lactis*	3259	2MI6	-	ND	ND	ND	19	0	19
*L*. *lactis*	3261	2BI3	-	ND	ND	ND	18	2	20
*L*. *lactis*	3260	2BI1	-	ND	ND	ND	17	3	20
*L*. *lactis*	3698	2BI2	-	ND	ND	ND	19	1	20
*L*. *lactis*	3262	2BII1	-	ND	ND	ND	17	2	19
*L*. *lactis*	-	2BII3 = 2BI3	-	ND	ND	ND	18	2	20

Phenotypic characterization of strains was complemented by determining the ability to produce EPS on media supplemented with 10% sucrose. According to our results, only strains belonging to *Weissella* sp. and *Leuconostoc* sp. produced slimy colonies and were characterized as EPS producing (Table 2).

Strains identified as *L*. *plantarum*, *L*. *brevis* and *Weissella* sp. were also analysed in terms of their antagonistic activity against spore-forming, bread spoilage bacteria (Table 2). The antagonism of LAB was strain dependent. The strongest antagonistic activity was observed against *B*. *brevis* and the weakest, against *L*. *fusiformis*.

### Development of starter cultures

Selection of LAB species for starter cultures was based on the literature data on the biodiversity of backslopped sourdoughs [21] and our recent results of the microbial diversity and the community growth dynamics in spontaneous wholegrain sourdoughs [7]. A meta-analysis of several hundred backslopped sourdoughs revealed that the two most prevalent groups of LAB were the *Fructilactobacillus sanfranciscensis* [basonym: *Lactobacillus sanfranciscensis*] (present in 47% of the sourdoughs), and the *L*. *plantarum* group (present in 44% of the sourdoughs). The *L*. *brevis* group, the *Limosilactobacillus reuteri* [basonym: *Lactobacillus reuteri*] group, and the *Companilactobacillus alimentarius* [basonym: *Lactobacillus alimentarius*] group were identified in 22%, 19%, and 18% of the examined sourdoughs, respectively. According to our studies, in all wholegrain sourdough samples obtained after 48 hr and 72 hr, the dominant population of LAB was lactobacilli. From the above-mentioned prevalent LAB groups only representants of *L*. *plantarum* and *L*. *brevis* species were isolated and therefore the strains belonging to these species were used for starter cultures. The *L*. *plantarum* and *L*. *brevis* were also the most frequently isolated types of lactobacilli from rye sourdoughs [7]. It is worth to note that *L*. *plantarum*—a homofermentative LAB—produces high acidity in all vegetable fermentations and plays the major role also in the cereal fermentation [46]. The analysis performed by Van Kerrebroeck and co-workers [22] showed that the next prevalent species were *Leuconostoc*, *Pediococcus*, and *Weissella*. Other species of LAB were present in 10% or less of the sourdoughs. Comparison of the rye sourdough samples obtained at three time points showed that after 24 hr of fermentation, the abundance of *Weissella* was higher than lactobacilli; however, as fermentation progressed, its abundance decreased in favour of lactobacilli [7]. Based on the abundance of *Weissella* at the early stage of fermentation a strain belonging to this species was also selected for developing starter cultures.

Selection of strains representing *L*. *plantarum*, *L*. *brevis* and *Weissella* sp. was made on the basis of the genotypic and phenotypic characterization of isolates. The acidifying *L*. *plantarum* 2MI8 and 6PIII6B, strains used in the presented in this work starter cultures, represent different clusters in RAPD-PCR analysis. One of the most important criteria for selecting strains was the antagonistic activity against spore-forming bacteria responsible for a spoilage process of bakery products known as ropiness or rope. Some rope-producing microorganisms such as toxigenic *B*. *cereus* can also pose safety risks for consumers and have been implicated in foodborne outbreaks [47]. Sporeformers are present in raw materials such as flours, yeast, or bread additives as well as on processing equipment [48]. Spore-forming bacteria contaminating flours originate from cereals: they are generally concentrated in the outer layers of grains and thus are particularly associated with the wholegrain flours [45, 46]. Microbiological spoilage affects bread quality causing heavy economic losses and in the case of bacteria even the potential presence of one spore may lead to food spoilage during bread storage in environmental conditions favourable to the germination and outgrowth of these spore-forming microorganisms. The development of starter cultures with strains having antagonistic activity against bread spoilage bacteria can help to assure the microbiological quality and safety of bakery products [49]. The analysis of antagonistic activity against spore-forming bacteria allowed for selection of strains that can reduce spoilage of bread. The 6PIII6B strain is characterized by the strongest antagonistic activity against two strains of spore-forming, food spoilage bacteria: *B*. *brevis* 6LIII2 and *L*. *fusiformis* 6LI1. The 2MIII4 strain is one of two *L*. *brevis* strains with the highest antagonistic activity against *B*. *brevis* 6LIII2. Selection of *Weissella* 6PI3 was based on its antagonistic action toward *B*. *cereus* 6LII1 and *B*. *brevis* 6LIII2. Moreover, the *Weissella* 6PI3 strain is characterized by the ability to produce EPS, further confirmed by our recent publication [50] and the highest spectrum of catabolized carbon sources among other *Weissella* strains. *Weissella* strains have been used for *in situ* EPS synthesis to improve the texture and quality of breads. It has been reported that microbial *in situ* production of EPS in sourdoughs is more effective than the addition of comparable amounts of EPS during the production of bread dough [51]. Furthermore, produced EPS have potential gut health-promoting properties. Intestinal microbes can metabolize these compounds that have been shown to possess prebiotic properties [13]. Another advantage of our starter cultures is that the strains have been isolated from the wholemeal sourdoughs to which they are dedicated. Ventimiglia and co-workers [52] showed that autochthonous strains harbouring interesting metabolic traits might be competitive towards other autochthonous species or strains isolated from a sourdough environment different from that used for bread production, therefore the origin of strains is important for composition of starter cultures. Furthermore, the use of such strains results in better quality of bread, its original sensory properties and allows the industry to avoid the uniformity of taste and smell of baked goods. When designing bread starter cultures, different species and strains should be taken into account in order to prevent the depletion of the taste and aroma characteristics of the bread and its sameness [4]. These criteria were met by starter cultures developed under this study: XI, XII and XIIB composed of different sets of LAB including various bacteria at a species and strain level (Table 1). These starter cultures were designed in a way to ensure that the constituent strains do not inhibit their own growth. In order to obtain starter cultures, the biomass of bacterial strains was mixed in appropriate proportions, according to description in Table 1, assuming that 1 ml of the starter culture should contain 1x10^10^ CFU.

### Evaluation of starter cultures for sourdough fermentation

**Fig 1:**
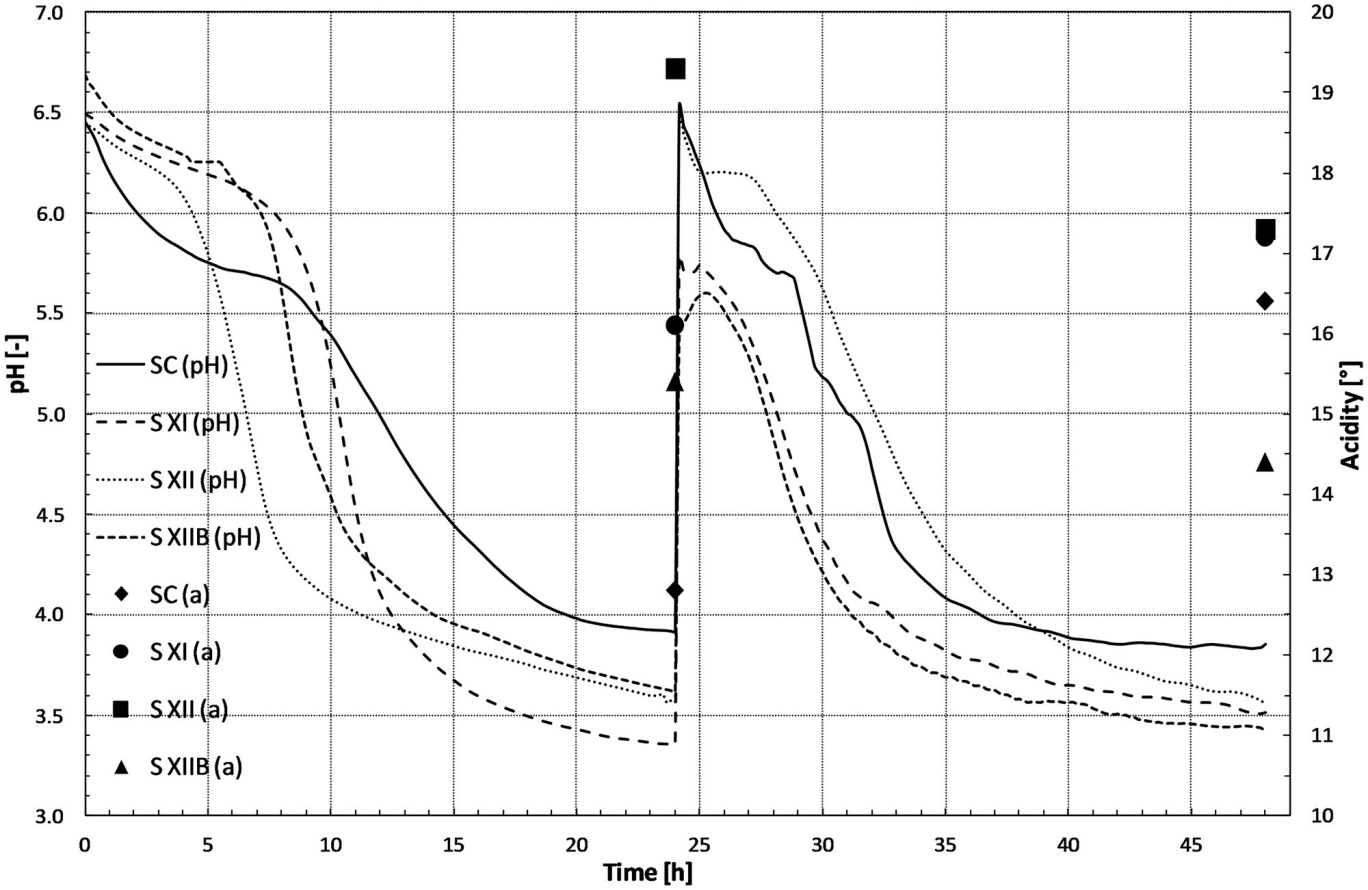
Active and potential acidity changes during sourdough fermentation. Active acidity (pH) and potential acidity (a).

Rye flour applied in this research was suitable for wholegrain rye bread production [26]. The active acidity (pH) was monitored during the sourdough preparation, as well as the potential acidity, and the results are shown in Fig 1. After 48 hr of the sourdough fermentation, the lowest potential acidity (14.4 Acidity degree) and at the same time the lowest pH (3.42) was observed for XIIB starter culture initiated sourdough, while the other sourdoughs were characterized by higher potential acidity (17.3 Acidity degree) and similar pH—3.53 on average. Sourdough obtained using the commercial LV2 starter culture showed a potential acidity of 16.4 Acidity degree, lower than for SXI and SXII sourdoughs, but higher than SXIIB one, and the highest pH (3.85) among the investigated sourdoughs.

### Evaluation of bread produced with selected sourdoughs

Based on the selected sourdoughs the breads were manufactured. The raw data of the industrially produced wholemeal rye bread evaluation are deposited to a public repository (http://eprints.ibb.waw.pl/2105). In the recipes for bread, regardless of the addition of yeast, the share of flour used in the form of sourdough was always the same. Dough without yeast addition was longer fermented than those prepared with contribution of yeast (240 min. vs 90 min.).

The volume of rye breads (Table 3) made only with sourdough (BS) based on cultures XI, XII and XIIB was similar (815, 817 and 814 cm^3^, respectively) and statistically significantly higher as compared to the volume of bread manufactured with the use of commercial culture (BSC)—781 cm^3^. For all yeast supplemented breads (BSY), a significantly larger volume was observed than for sourdough based only breads (BS)—an average of 50 cm^3^. Among BSY, commercial culture bread (BSCY) had a similar volume (849 cm^3^) as the volume of sourdough bread derived from the tested starter cultures, i.e. BSXIY (847 cm^3^) and BSXIIY (853 cm^3^), while the use of culture XIIB gave a more favourable effect on this parameter, because breads obtained with the use of this culture (BSXIIBY) had the largest volume (886 cm^3^), compared to all tested breads. As shown in Table 3, the volume of bread was influenced both by the microorganisms composition in sourdough, and also by the yeast application in the rye dough. The use of XIIB culture seems to be particularly beneficial. A statistically significantly higher volume of bread loaves was obtained using sourdough derived from this culture, as compared to other ones. This effect can be attributed to the presence of *Weissella confusa*/*cibaria*, that produce EPS and/or the action of yeast (added to dough or derived from flour). However, XII starter culture consisting of four bacterial strains, including *Weissella confusa*/*cibaria*, did not show a similar effect, which should be attributed to the yeast—bacteria interaction in sourdough and dough, which could have a strain rather than species character [4]. In addition, the lower acidity of the sourdough obtained using XIIB culture, compared to XII (Fig 1), resulted in better yeast fermentation, which resulted in a larger bread loaf volume. In conclusion, it can be stated, that statistically the largest volume among the all tested breads loaves was observed for those obtained using XIIB culture origin sourdough, especially baked with the addition of yeast (BSXIIBY).

**Table 3:** Quality features of the investigated bread.

SC*	BT	Bread	Weight [g]	Volume [cm^3^]	Baking loss [%]	Organoleptic assessment		Crumb moisture [%]		Crumb hardness [N]		Crumb TTA [°]
						Score	QC	0	5th	0	5th	
**C**			519±7^ab^**	815±37^c^	13.6±1.1^bc^	35.9±2.7^b^	I	48.1±1.9^c^	46.9±1.2^d^	45.9±8.8^a^	57.1±17.9^a^	7.0±2.3
**XI**			520±7^a^	831±21^b^	13.4±1.1^c^	36.2±2.1^b^	I	47.4±0.2^d^	47.6±0.8^c^	41.5±2.0^b^	56.6±5.3^a^	6.5±1.7
**XII**			516±5^b^	835±22^b^	14.1±0.9^b^	37.0±2.2^ab^	I	49.1±1.2^b^	48.7±0.6^b^	31.0±1.7^c^	41.0±5.5^b^	8.0±2.3
**XIIB**			507±7^c^	850±39^a^	15.5±1.1^a^	37.7±0.9^a^	I	50.4±0.8^a^	50.0±0.9^a^	23.6±2.2^d^	33.0±3.8^c^	6.5±1.7
	**BS**		518±8^a^	807±19^b^	13.7±1.3^b^	36.2±2.0^a^	I	47.9±1.3^b^	47.5±1.4^b^	37.7±11.4^a^	53.6±15.1^a^	8.8±0.9
	**BSY**		512±7^b^	859±20^a^	14.6±1.2^a^	37.0±2.4^a^	I	49.6±1.5^a^	49.1±1.2^a^	33.3±8.6^b^	40.3±9.1^b^	5.3±0.5
**C**		**BSC**	523±5^a^	781±14^d^	12.8±0.8^d^	35.9±1.9^bc^	I	46.4±0.0^g^	45.9±0.1^f^	53.5±0.4^a^	72.5±1.1^a^	9.0±0.0
**C**		**BSCY**	514±5^b^	849±12^b^	14.4±0.9^c^	35.8±3.4^bc^	I	49.8±0.0^c^	48.0±0.^d^	38.3±1.6^c^	41.7±4.0^de^	5.0±0.0
**XI**		**BSXI**	523±5^a^	815±1^c^	12.9±0.9^d^	36.3±2.1^abc^	I	47.6±0.0^e^	46.8±0.1^e^	39.9±0.5^c^	60.3±5.5^b^	8.0±0.0
**XI**		**BSXIY**	517±7^b^	847±17^b^	13.9±1.1^c^	36.0±2.2^abc^	I	47.3±0.0^f^	48.3±0.1^c^	43.1±1.3^b^	52.9±0.5^c^	5.0±0.0
**XII**		**BSXII**	518±4^ab^	817±13^c^	13.7±0.7^cd^	35.4±2.0^a^	II	48.1±0.1^d^	48.1±0.1^cd^	32.4±0.4^d^	45.5±3.0^d^	10.0±0.0
**XII**		**BSXIIY**	514±6^bc^	853±11^b^	14.4±0.9^bc^	38.1±1.6^a^	I	50.2±0.1^b^	49.2±0.1^b^	29.7±1.1^d^	36.5±1.1^ef^	6.0±0.0
**XIIB**		**BSXIIB**	508±6^cd^	814±9^c^	15.3±0.9^ab^	37.6±1.0^ab^	I	49.7±0.0^c^	49.3±0.1^b^	24.8±2.4^e^	36.1±2.2^ef^	8.0±0.0
**XIIB**		**BSXIIBY**	506±8^d^	886±10^a^	15.7±1.3^a^	37.8±0.8^ab^	I	51.1±0.0^a^	50.8±0.0^a^	22.3±1.8^e^	29.9±0.3^f^	5.0±0.0

*SC–starter culture, BT–baking technology, QC–quality class, 0 –at day of baking, 5^th^–at 5^th^ day of storage, TTA–total titratable acidity expressed in terms Acidity degree.

**Average ± SD; values within the column section denoted with the same superscript are not statistically different according to Duncan test (α ≤ 0.05).

It was observed the influence both of the starter culture applied, as well as yeast on the crumb moisture and texture of the investigated breads. Breads obtained with the use of sourdough derived from the starter culture XII and XIIB, regardless of the yeast addition, were characterized by greater crumb moisture, and lower crumb hardness on the day of baking and after 5 days of storage, as compared to bread based on C and XI starter culture (Table 3). Breads obtained with the use of starter culture XIIB were characterized by the highest crumb moisture and the lowest crumb hardness. In addition, all breads prepared with yeast addition were characterized by higher moisture and lower crumb hardness, both on the day of baking and after storage, as compared to breads made without yeast addition. Storage period did not significantly affect the crumb moisture of the tested breads, while the crumb hardness of all tested breads increased during 5-day storage, which was associated with the starch retrogradation process [53, 54]. However, this process took place with varying intensity, depending on the starter culture applied and the yeast presence. In yeast prepared bread, the crumb hardness increased by an average of 7 N, and in the case of breads made with the use of sourdough alone by 16 N. In case of breads made with the use of starter cultures XII and XIIB, regardless of the use of yeast, it was observed the smallest increase in hardness. This is probably due to the 25% participation of *Weissella confusa*/*cibaria* 6PI3 in these cultures. It has been proved that these bacteria have a high ability to produce EPS [55], and their presence influences the texture and rheological properties of cereal-based beverages and sourdough bread [56], usually allows to delay the bread aging [57, 58]. Katina et al. [59] proved, that *Weissella confusa* produces a significant amount of EPS, i.e. dextran and isomaltosaccharides in wheat dough, without causing strong dough acidification. On the other hand, sourdoughs in which significant, from technological point of view, quantities of dextran are produced during fermentation due to *Leuconostoc mesenteroides* action, are commonly used as bread improvers [60].

Table 3 also contains the results of the crumb acidity analysis. Regardless of the starter culture used for the sourdough production, bread crumb with yeast addition has on average less than 3 degrees of TTA. It can be associated with a much shorter (by 150 min.) time of yeast dough fermentation. The use of XII starter culture based sourdough without yeast addition allows to obtain bread with highest crumb acidity (10 Acidity degree), while with the use of culture XI and XIIB—the lowest (6 Acidity degree). The use of LV2-SC commercial culture sourdough resulted in the intermediate crumb acidity– 9 Acidity degree. The crumb acidity did not have a significant effect on the bread organoleptic assessment. The lowest consumer acceptance was noted for BSXII bread, while BSXIIY and BSXIIB and BSXIIBY ones obtained the highest score (Table 3), i.e. the same breads that had the lowest crumb hardness.

The taste of the evaluated breads depended not only on their crumb acidity, but also on the content of selected organic acids in these breads. Worse rated BSXII sourdough bread contained the highest amount of acetic acid among breads based on newly created starter cultures without yeast addition (Table 4), but due to a significant amount of lactic acid it had an ideal ratio of lactic acid to acetic acid, equal to 75:25 (Table 4). Breads well scored by panellists, i.e. BSXIIB and BSXIIBY, were distinguished by a significantly lower content of acetic acid, as compared to other breads, and a ratio of lactic acid to acetic acid: 96:4 and 91:9, respectively. BSXIIY bread, best evaluated in organoleptic assessment, although it contained almost three times as much acetic acid as compared to BSXIIB and BSXIIBY bread, but it had also an almost ideal ratio of lactic acid to acetic acid: 76:24 (Table 4). Based on the obtained results, it can be concluded, that the taste of the evaluated breads was mainly influenced by the acetic acid content, and to a lesser extent by the ratio of the two dominant acids, i.e. lactic and acetic. Similar conclusion was drawn by Gänzle [61]. Among the remaining organic acids, the content of propionic acid deserves a special attention, the highest in XI starter culture sourdough breads (BSXI and BSXIY). As it is commonly known, propionic acid has a preservative effect on bread crumb during storage and, like acetic and lactic acids, has a certain ability to weaken the response to postprandial blood glucose and insulin levels [62].

**Table 4:** Organic acids content in the investigated breads (%).

SC*	BT	Bread	Tartaric	Citric	Malic	Lactic	Acetic	Propionic	Sum of organic acids	Lactic + acetic	Lactic% in lactic+acetic
**C**			0.008±0.001^a^**	0.007±0.002^a^	0.024±0.007^b^	0.311±0.081^c^	0.136±0.029^a^	0.128±0.012^b^	0.614±0.103^b^	0.447±0.11^b^	69.4±1.3^d^
**XI**			0.003±0.004^b^	0.002±0.001^c^	0.045±0.005^a^	0.452±0.112^a^	0.067±0.012^c^	0.154±0.004^a^	0.723±0.123^a^	0.519±0.124^a^	86.8±0.9^b^
**XII**			0.009±0.001^a^	0.003±0.001^b^	0.014±0.009^c^	0.336±0.108^b^	0.111±0.041^b^	0.075±0.01^c^	0.548±0.139^c^	0.447±0.149^b^	75.3±1.2^c^
**XIIB**			0.004±0.004^b^	0.003±0.001^b^	0.023±0.003^bc^	0.364±0.119^b^	0.023±0.003^d^	0.076±0.007^c^	0.492±0.127^d^	0.387±0.116^c^	93.4±2.7^a^
	**B**		0.008±0.001^a^	0.002±0.002^a^	0.025±0.014^a^	0.456±0.066^a^	0.101±0.06^a^	0.108±0.035^a^	0.701±0.09^a^	0.558±0.055^a^	82.1±10.9^a^
	**BSY**		0.004±0.005^b^	0.005±0.003^b^	0.028±0.012^a^	0.275±0.05^b^	0.068±0.033^b^	0.108±0.04^a^	0.488±0.097^b^	0.343±0.05^b^	80.4±9.4^b^
**C**		**BSC**	0.008±0.000^a^	0.006±0.000^b^	0.025±0.011^b^	0.381±0.01^d^	0.161±0.001^a^	0.122±0.004^b^	0.703±0.003^b^	0.542±0.011^c^	70.3±0.4^e^
**C**		**BSCY**	0.009±0.001^a^	0.009±0.001^a^	0.023±0.005^b^	0.241±0.009^f^	0.111±0.002^c^	0.134±0.016^b^	0.526±0.016^d^	0.352±0.007^f^	68.5±1.3^e^
**XI**		**BSXI**	0.006±0.003^a^	0.000±0.000^f^	0.043±0.000^a^	0.548±0.011^a^	0.078±0.003^d^	0.154±0.005^a^	0.83±0.017a	0.626±0.015^a^	87.6±0.2^c^
**XI**		**BSXIY**	0.000±0.000^b^	0.003±0.000^d^	0.047±0.007^a^	0.355±0.008^e^	0.057±0.001^e^	0.154±0.005^a^	0.617±0.004^c^	0.412±0.006^e^	86.1±0.5^c^
**XII**		**BSXII**	0.009±0.001^a^	0.002±0.000^e^	0.007±0.005^c^	0.43±0.011^c^	0.146±0.008^b^	0.075±0.013^c^	0.668±0.019^b^	0.576±0.003^b^	74.6±1.6^d^
**XII**		**BSXIIY**	0.009±0.002^a^	0.004±0.000^cd^	0.021±0.006^b^	0.243±0.002^f^	0.076±0.001^d^	0.076±0.011^c^	0.429±0.011^e^	0.319±0.003^g^	76.1±0.0^d^
**XIIB**		**BSXIIB**	0.007±0.000^a^	0.001±0.000^e^	0.025±0.003^b^	0.466±0.02^b^	0.021±0.003^f^	0.082±0.004^c^	0.602±0.024^c^	0.487±0.017^d^	95.8±0.7^a^
**XIIB**		**BSXIIBY**	0.000±0.000^b^	0.004±0.000^c^	0.022±0.003^b^	0.261±0.007^f^	0.026±0.002^f^	0.07±0.001^c^	0.383±0.006^f^	0.287±0.009^h^	91.1±0.4^b^

*SC–starter culture, BT–baking technology.

**Average ± SD; values within the column section denoted with the same superscript are not statistically different according to Duncan test (α ≤ 0.05).

As it results from the data contained in the S2 Table in all investigated breads, the higher glucose and maltose content was determined in breads prepared without yeast addition, than in those prepared with such addition. The type of the applied starter culture influenced the content of simple sugars in the evaluated breads, however, it seems that the taste of these breads depended more on the amount of acetic and lactic acids (Table 4) than on the sugar content.

The basic chemical composition of investigated breads was similar in terms of content: ashes, dietary fibre (DF) and protein (Table 5). The exception was the definitely higher protein content in sourdough breads BSXIIB and BSXIIBY (applied XIIB starter culture) as compared to other breads. As can be concluded from data given in Table 5, the protein and ash content in the investigated breads did not depend on baking technology, but more on the type of starter culture used in sourdough preparation, and thus on the different pH of these sourdoughs. As previously mentioned, the application of XIIB culture caused in sourdough decreased acidity as compared to other breads, resulting in better yeast fermentation, and also greater degradation of carbohydrates in bread based on this sourdough only, or the sourdough and yeast addition. The intensive fermentation of the dough with the XIIB starter culture resulted in the largest bread volume and the highest utilization of carbohydrates by yeast, which at the same time resulted in a percentage increase in the protein and ash content of such breads. Probably, as mentioned above, in the dough with the XIIB culture, it was possible to obtain the best interaction of yeast with bacteria contained in the sourdough. The confirmation of the greatest loss of carbohydrates in these breads is also the greatest baking loss observed during the baking of BSIIB and BSXIIBY breads. It can be also seen a greater amount of total dietary fibre (TDF) and its water insoluble fraction (IDF) in all bread with the yeast addition, although these breads were characterized by a lower crumb hardness in relation to the other breads (prepared without yeast addition). The higher content of IDF fraction in breads with the addition of yeast was caused by greater amylose retrogradation, and the formation of resistant starch fraction RS_3_ [63]. As is known, the predominance of LAB during dough fermentation restricts the starch retrogradation process in bread [59].

**Table 5:** Basic composition of the investigated breads [g 100g^-1^ db].

SC*	BT	Bread	Protein [Nx6.25]	Ash	Dietary fibre		
					Insoluble fraction	Soluble fraction	Total
**C**			8.46±0.01^c^**	3.67±0.02^c^	11.95±0.58^a^	4.4±0.41^b^	16.35±0.18^b^
**XI**			8.43±0.09^c^	3.64±0.02^c^	11.96±0.43^a^	4.64±0.52^a^	16.59±0.12^a^
**XII**			8.57±0.06^b^	3.73±0.03^b^	11.8±0.36^b^	4.45±0.07^b^	16.24±0.34^bc^
**XIIB**			10.06±0.14^a^	3.81±0.02^a^	11.46±0.52^c^	4.74±0.13^a^	16.21±0.65^c^
	**BS**		8.86±0.67^a^	3.71±0.07^a^	11.38±0.24^b^	4.74±0.24^a^	16.12±0.41^b^
	**BSY**		8.9±0.79^a^	3.72±0.07^a^	12.2±0.22^a^	4.38±0.33^b^	16.58±0.13^a^
**C**		**BSC**	8.46±0.01^cd^	3.67±0.02^cd^	11.45±0.08^e^	4.75±0.05^bc^	16.2±0.03^d^
**C**		**BSCY**	8.46±0.01^cd^	3.66±0.01^d^	12.46±0.03^a^	4.05±0.11^f^	16.51±0.08^c^
**XI**		**BSXI**	8.43±0.15^d^	3.64±0.02^d^	11.59±0.09^d^	5.09±0.04^a^	16.67±0.05^ab^
**XI**		**BSXIY**	8.42±0.05^d^	3.65±0.02^d^	12.33±0.05^a^	4.19±0.08^f^	16.51±0.12^c^
**XII**		**BSXII**	8.61±0.05^c^	3.71±0.01^c^	11.49±0.05^cd^	4.48±0.09^de^	15.96±0.13^d^
**XII**		**BSXIIY**	8.53±0.02^cd^	3.76±0.01^b^	12.11±0.00^b^	4.42±0.07^e^	16.53±0.07^c^
**XIIB**		**BSXIIB**	9.94±0.03^b^	3.81±0.02^a^	11.01±0.06^f^	4.63±0.02^cd^	15.64±0.08^e^
**XIIB**		**BSXIIBY**	10.18±0.05^a^	3.81±0.02^a^	11.91±0.03^c^	4.86±0.03^b^	16.77±0.06^a^

*SC–starter culture, BT–baking technology.

**Average ± SD; values within the column section denoted with the same superscript are not statistically different according to Duncan test (α ≤ 0.05).

Dough prepared with the use of starter culture only (without the yeast addition) resulted in a significantly reduced the sum of myo-inositol phosphates in breads, i.e. from 1.5 to approximately 5 times as compared to the applied raw material (Table 6), i.e. wholemeal rye flour, in which the total content of inositol phosphates was 1.18% db. The smallest amount of inositol hexakisphosphate (IP_6_) was determined in a bread BSXI and BSXII—0.07 and 0.03%. of bread db. The content of lower inositol phosphates (IP_5_—IP_2_), calculated as IP sum minus IP_6_ (Table 6), was also the lowest in these breads—0.21 and 0.22% bread db, while in bread based on commercial starter culture C (BSC) this amount was 0.34% db, and in BSXIIB—0.52% db. The use of starter cultures XI and XII for sourdough preparation resulted in a greater reduction in the content of IP_6_ in bread, as compared to the use of commercial culture C (Table 6). This is the effect of greater acidity of sourdoughs obtained with the use of XI and XII starter cultures, compared to the C commercial culture (Fig 1). However, the worst effect, i.e. the least degradation of IP_6_ into lower phosphate derivatives, was observed for XIIB culture obtained sourdough, which also characterized by the lowest acidity of 14.4 Acidity degree (Fig 1). Phytic acid (IP6) is known as a food inhibitor which chelates micronutrient. They become less bioavailabe for monogastric animals, including humans, due to the lack of the phytase enzyme in their digestive tracts. The lower forms of inositol phosphates have a lower binding capacity for metals like iron and zinc [14]. Thus, food products with reduced amounts of IP6 have improved nutritional quality.

**Table 6:** Myo-inositol phosphate content in breads [%, db].

SC*	BT	Bread	IP_6_	IP sum	Detected myo-inositol P
**C**			0.23±0.16^b^**	0.56±0.15^b^	
**XI**			0.2±0.16^b^	0.46±0.21^c^	
**XII**			0.11±0.10^c^	0.37±0.14^d^	
**XIIB**			0.36±0.10^a^	0.72±0.1^a^	
	**BS**		0.12±0.10^b^	0.44±0.23^b^	
	**BSY**		0.34±0.10^a^	0.62±0.08^a^	
**C**		**BSC**	0.1±0.00^e^	0.44±0.00^c^	IP_6_, IP_5_, IP_4_, IP_3_, IP_2_
**C**		**BSCY**	0.37±0.02^b^	0.69±0.01^b^	IP_6_, IP_5_, IP_4_, IP_3_, IP_2_
**XI**		**BSXI**	0.07±0.00^ef^	0.28±0.00^d^	IP_6_, IP_5_, IP_4_, IP_3_, IP_2_
**XI**		**BSXIY**	0.33±0.05^cd^	0.63±0.08^b^	IP_6_, IP_5_, IP_4_, IP_3_, IP_2_
**XII**		**BSXII**	0.03±0.00^f^	0.25±0.02^d^	IP_6_, IP_5_, IP_4_, IP_3_, IP_2_
**XII**		**BSXIIY**	0.2±0.01^d^	0.5±0.03^c^	IP_6_, IP_5_, IP_4_, IP_3_, IP_2_
**XIIB**		**BSXIIB**	0.28±0.02^d^	0.8±0.08^a^	IP_6_, IP_5_, IP_4_, IP_3_, IP_2_
**XIIB**		**BSXIIBY**	0.44±0.01^a^	0.64±0.01^b^	IP_6_, IP_4_, IP_3_, IP_2_

*SC–starter culture, BT–baking technology.

**Average ± SD; values within the column section denoted with the same superscript are not statistically different according to Duncan test (α ≤ 0.05).

Wholemeal rye flour dough fermented with the participation of sourdoughs prepared from starter cultures, with the yeast addition (BSY), resulted in a much smaller degradation of higher inositol phosphates to lower phosphate derivatives (Table 6), about 2–2.5 times as compared to the applied raw material, i.e. wholemeal flour. Certainly, this was influenced by a much shorter dough fermentation time when yeast were applied (90 min.) compared to the dough prepared with sourdough use only (240 min.).

## Conclusions

LAB isolated from spontaneous sourdoughs made from wholemeal rye flour, were used to develop starter cultures for bread production. All investigated new starter cultures based on selected LAB strains, proved to be more suitable for sourdough and rye bread making in comparison to the LV2 commercial starter culture. The most suitable starter culture to produce wholemeal rye flour bread was XIIB one (best organoleptic score, highest moisture and lowest hardness of the crumb), containing *Lactobacillus plantarum* 2MI8 and *Weissella confusa*/*cibaria* 6PI3 strains. Fermentation of doughs prepared from wholemeal rye flour with the use of starter cultures resulted in a significant decrease of the content of higher myo-inositol phosphates in all rye breads, compared to the rye flour used, especially in breads prepared without yeasts addition.

It can be concluded that autochthonous LAB isolates with the promising technological properties were selected for starter cultures and the best one for baking rye wholemeal bread was selected. Considering that the bread produced using such starter cultures is more attractive for consumers due to its overall health promoting properties it might result in its increased demand and consumption. Before the use in the industry the proposed starter cultures containing *Weissella* strain should be further investigated in terms of safety aspects. Although, *Weissella* spp. are still not recognized as GRAS by the FDA neither as QPS by the EFSA, it does do not justify their rejection as commercial starters [64]. *Weissella* spp. are extensively involved in spontaneous fermented foods, especially fruits and vegetables based products, in which they could dominate the process. It is important to note that most of the *Weissella* strains with health-promoting properties have been shown to be safe, due to the absence of virulence or antibiotic-resistant genes. A large number of scientific studies continue to report on and to support the use of *Weissella* strains in the food and pharmaceutical industries [65].

## Supporting information

S1 Fig Results of genotyping the bacterial isolates from the spontaneous rye wholegrain sourdoughs obtained after the reaction of RAPD-PCR with B10 and GACA primers. (A) *L*. *plantarum*, B10. (B) *L*. *plantarum*, GACA. (C) *L*. *brevis*, B10. (D) *L*. *brevis*, GACA. (E) *C*. *crustorum*, B10. (F) *C*. *crustorum*, GACA. (G) *P*. *pentosaceus*, B10. (H) *P*. *pentosaceus*, GACA. (I) *Weissella* sp., B10. (J) *Weissella* sp., GACA. (K) *Leuconostoc* sp., B10. (L) *Leuconostoc* sp., GACA. (M) *L*. *lactis*, B10. (N) *L*. *lactis*, GACA.
(PDF)

S1 Table API 50 CHL results of the lactic acid bacteria (LAB) excluding *Enterococcus* strains, isolated from the rye wholegrain sourdoughs. (PDF)

S2 Table Glucose, maltose, glycerol and ethanol in the investigated breads [% db]. (PDF)

S1 File (PDF)
