# Supplementary material for: Developing lactic acid bacteria starter cultures for wholemeal rye flour bread with improved functionality, nutritional value, taste, appearance and safety
Source: PLoS One. 2022 Jan 14;17(1):e0261677. doi: 10.1371/journal.pone.0261677 (PMC8759695; doi:10.1371/journal.pone.0261677)
Supplement: S1 Fig — (A) L. plantarum, B10. (B) L. plantarum, GACA. (C) L. brevis, B10. (D) L. brevis, GACA. (E) C. crustorum, B10. (F) C. crustorum, GACA. (G) P. pentosaceus, B10. (H) P. pentosaceus, GACA. (I) Weissella sp., B10. (J) Weissella sp., GACA. (K) Leuconostoc sp., B10. (L) Leuconostoc sp., GACA. (M) L. lactis, B10. (N) L. lactis, GACA. (PDF) [file pone.0261677.s001.pdf]

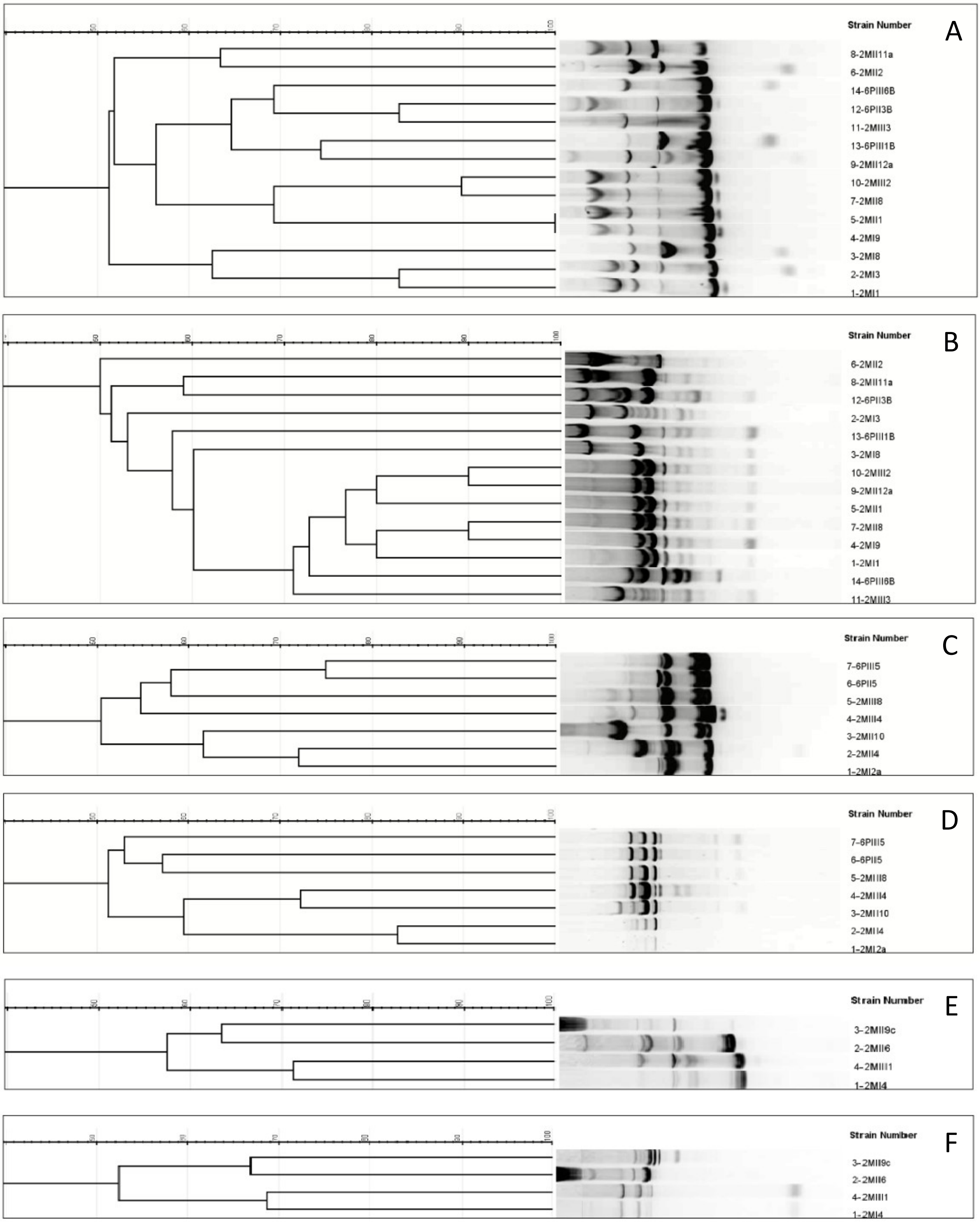

A
50
60
70
80
90
100
Strain Number
8-2MII11a
6-2MII2
14-6PIII6B
12-6PII3B
11-2MIII3
13-6PIII1B
9-2MII12a
10-2MIII2
7-2MII8
5-2MII1
4-2MI9
3-2MI8
2-2MI3
1-2MI1
B
Strain Number
6-2MII2
8-2MII11a
12-6PII3B
2-2MI3
13-6PIII1B
3-2MI8
10-2MIII2
9-2MII12a
5-2MII1
7-2MII8
4-2MI9
1-2MI1
14-6PIII6B
11-2MIII3
C
Strain Number
7-6PIII5
6-6PII5
5-2MIII8
4-2MIII4
3-2MII10
2-2MII4
1-2MI2a
D
Strain Number
7-6PIII5
6-6PII5
5-2MIII8
4-2MIII4
3-2MII10
2-2MII4
1-2MI2a
E
Strain Number
3-2MII9c
2-2MII6
4-2MIII1
1-2MI4
F
Strain Number
3-2MII9c
2-2MII6
4-2MIII1
1-2MI4

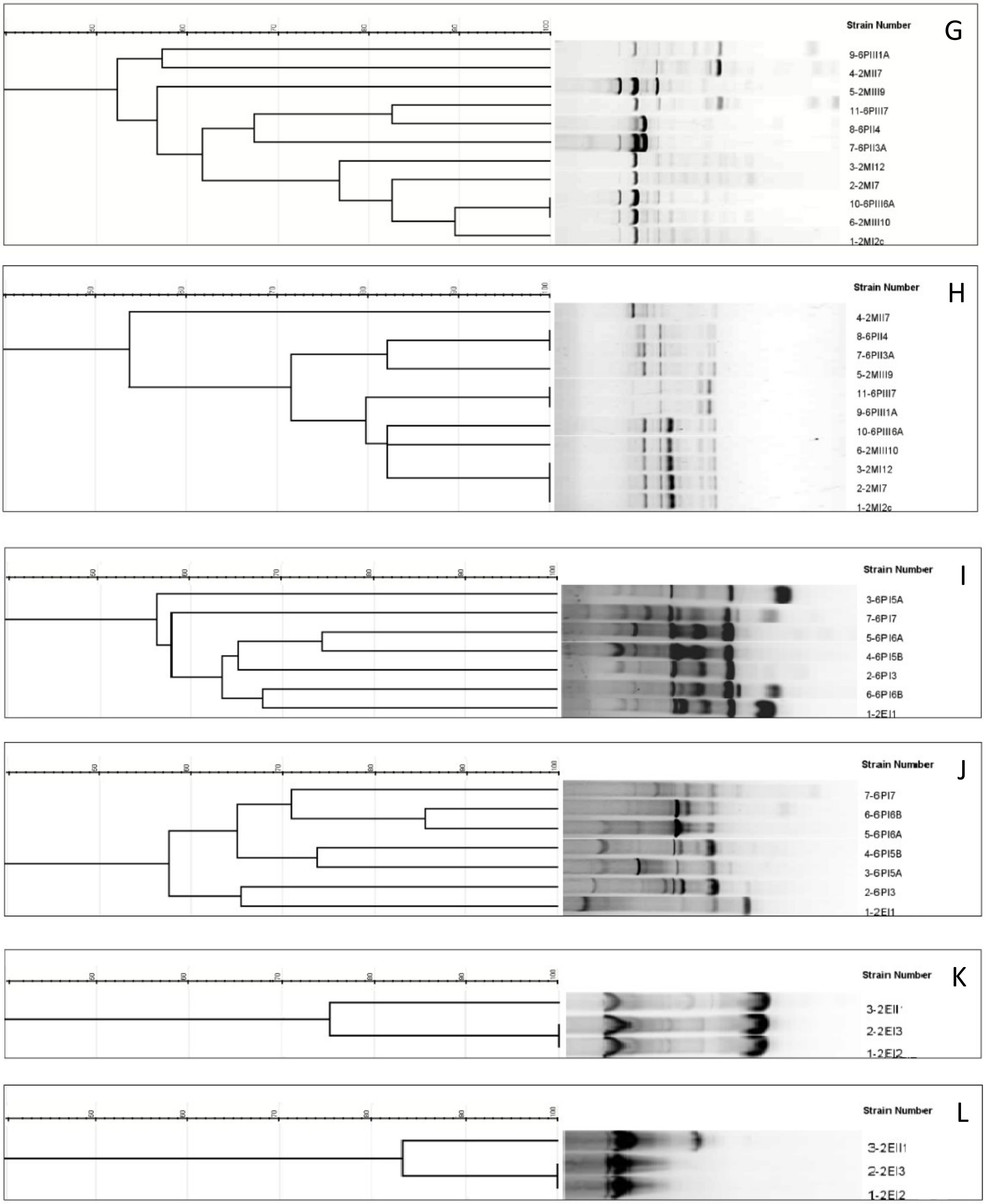

G
Strain Number
50
60
70
80
90
100
9-6PIII1A
4-2MII7
5-2MIII9
11-6PIII7
8-6PII4
7-6PII3A
3-2MI12
2-2MI7
10-6PIII6A
6-2MIII10
1-2MI2c
H
Strain Number
4-2MII7
8-6PII4
7-6PII3A
5-2MIII9
11-6PIII7
9-6PIII1A
10-6PIII6A
6-2MIII10
3-2MI12
2-2MI7
1-2MI2c
I
Strain Number
3-6PI5A
7-6PI7
5-6PI6A
4-6PI5B
2-6PI3
6-6PI6B
1-2EI1
J
Strain Number
7-6PI7
6-6PI6B
5-6PI6A
4-6PI5B
3-6PI5A
2-6PI3
1-2EI1
K
Strain Number
2-2EI3
1-2EI2
L
Strain Number
3-2EII1
2-2EI3
1-2EI2

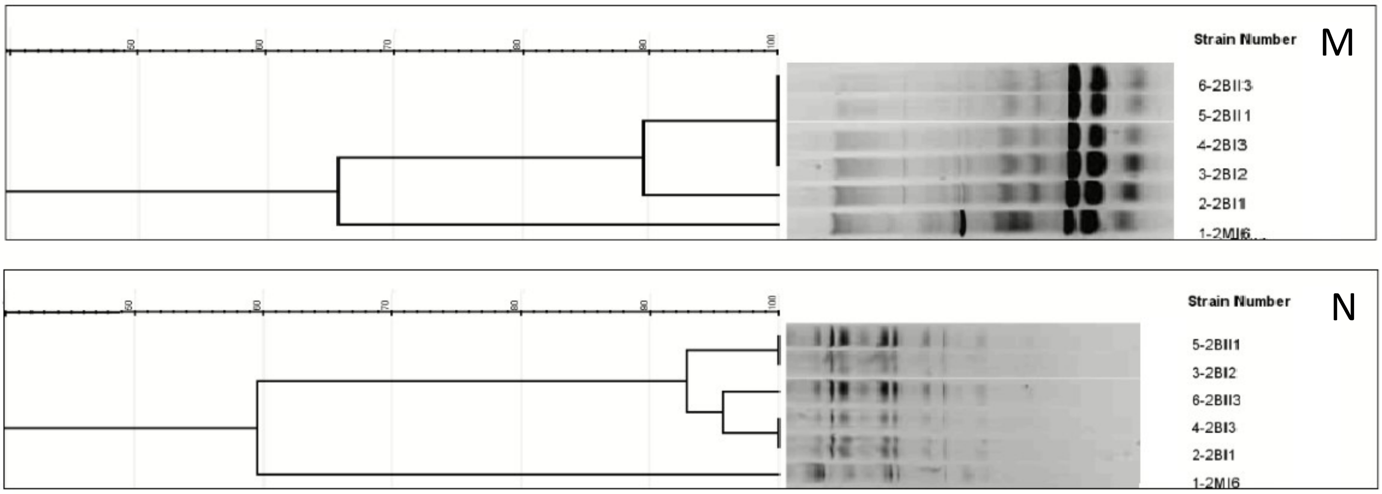

S1 Fig. Results of genotyping the bacterial isolates from the spontaneous rye wholegrain sourdoughs obtained after the reaction of RAPD-PCR with B10 and GACA primers. (A) *L. plantarum*, B10. (B) *L. plantarum*, GACA. (C) *L. brevis*, B10. (D) *L. brevis*, GACA. (E) *C. crustorum*, B10. (F) *C. crustorum*, GACA. (G) *P. pentosaceus*, B10. (H) *P. pentosaceus*, GACA. (I) *Weissella* sp., B10. (J) *Weissella* sp., GACA. (K) *Leuconostoc* sp., B10. (L) *Leuconostoc* sp., GACA. (M) *L. lactis*, B10. (N) *L. lactis*, GACA.
