# Supplementary material for: Developing lactic acid bacteria starter cultures for wholemeal rye flour bread with improved functionality, nutritional value, taste, appearance and safety
Source: PLoS One. 2022 Jan 14;17(1):e0261677. doi: 10.1371/journal.pone.0261677 (PMC8759695; doi:10.1371/journal.pone.0261677)
Supplement: S2 Table — (PDF) [file pone.0261677.s003.pdf]

S2 Table. Glucose, maltose, glycerol and ethanol in the investigated breads [% db]

SC*	BT	Bread	Maltose	Glucose	Glycerol	EtOH
**C**			0.59±0.04^{b**}	0.41±0.03^{b}	0.15±0.04^{b}	0.22±0.02^{b}
**XI**			0.72±0.18^{a}	0.52±0.13^{a}	0.17±0.03^{a}	0.23±0.04^{b}
**XII**			0.38±0.04^{c}	0.26±0.04^{d}	0.13±0.02^{c}	0.24±0.00^{b}
**XIIB**			0.73±0.18^{a}	0.35±0.13^{c}	0.14±0.01^{b}	0.26±0.01^{a}
	**B**		0.68±0.22^{a}	0.46±0.12^{a}	0.14±0.02^{b}	0.22±0.02^{b}
	**BSY**		0.51±0.12^{b}	0.31±0.08^{b}	0.15±0.04^{a}	0.25±0.02^{a}
**C**		**BSC**	0.55±0.01^{c}	0.44±0.01^{c}	0.11±0.00^{c}	0.21±0.02^{d}
**C**		**BSCY**	0.63±0.00^{b}	0.38±0.00^{d}	0.18±0.00^{a}	0.24±0.00^{c}
**XI**		**BSXI**	0.87±0.01^{a}	0.63±0.00^{a}	0.15±0.01^{b}	0.20±0.00^{d}
**XI**		**BSXIY**	0.56±0.00^{c}	0.40±0.00^{d}	0.19±0.01^{a}	0.27±0.01^{ab}
**XII**		**BSXII**	0.42±0.01^{d}	0.31±0.01^{e}	0.15±0.01^{b}	0.23±0.00^{c}
**XII**		**BSXIIY**	0.35±0.02^{e}	0.23±0.01^{f}	0.11±0.00^{c}	0.24±0.00^{c}
**XIIB**		**BSXIIB**	0.88±0.02^{a}	0.46±0.02^{b}	0.14±0.01^{b}	0.25±0.01^{bc}
**XIIB**		**BSXIIBY**	0.57±0.02^{c}	0.24±0.01^{f}	0.15±0.02^{b}	0.27±0.01^{a}

*SC – starter culture, BT – baking technology

**Average ± SD; values within the column section denoted with the same superscript are not statistically different according to Duncan test ($\alpha \leq 0.05$)
