# Supplementary material for: Developing lactic acid bacteria starter cultures for wholemeal rye flour bread with improved functionality, nutritional value, taste, appearance and safety
Source: PLoS One. 2022 Jan 14;17(1):e0261677. doi: 10.1371/journal.pone.0261677 (PMC8759695; doi:10.1371/journal.pone.0261677)
Supplement: S1 Table — (PDF) [file pone.0261677.s002.pdf]

S1 Table. API 50 CHL results of the lactic acid bacteria (LAB) excluding *Enterococcus* strains, isolated from the rye wholegrain sourdoughs.

Groups identified based on sequencing of 16S rRNA genes	IBB PAS culture collection no.	Strain	Negative control	Glycerol	Erythritol	D-arabinose	L-arabinose	D-ribose	D-xylose	L-xylose	D-adonitol	β methyl-D-xyloside	D-galactose	D-glucose	D-fructose	D-mannose	L-sorbose	L-rhamnose	Dulcitol	Inozitol	D-mannitol	D-sorbitol	α methyl-D-mannoside	α methyl-D- glucoside	N-acetyl-glucosamine	Amygdalin	Arbutin	Esculin	Salicin	D-cellobiose	D-maltose	D-lactose	D-melibiose	D-sucrose	D-trehalose	Inulin	D-melezitose	D-raffinose	Starch	Glycogen	Xylitol	β-gentiobiose	D-turanose	D-lyxose	D-tagatose	D-fucose	L-fucose	D-arabitol	L-arabitol	Gluconate	2-keto-gluconate	5-keto-gluconate
L. plantarum	3247	2MI1					+	+					+	+	+	+					+		+		+	+	+	+	+	+	+	+	+	+	+		+	+				+	+					+		+		
L. plantarum	3248	2MI3					+	+					+	+	+	+					+	+			+	+	+	+	+	+	+	+	+	+	+			+				+						+/-		+/-		
L. plantarum	3249	**2MI8**					+	+					+	+	+	+					+				+	+	+	+	+	+	+	+	+	+	+		+	+				+										
L. plantarum	3690	2MI9					+	+					+	+	+	+					+	+	+		+	+	+	+	+	+	+	+	+	+	+		+	+				+	+							+/-		
L. plantarum	3691	2MII1					+	+					+	+	+	+					+		+		+	+	+	+	+	+	+	+	+	+	+		+	+				+	+					+/-		+/-		
L. plantarum	3250	2MII2					+	+	+				+	+	+	+					+	+	+	+	+	+	+	+	+	+	+	+	+	+	+		+	+				+	+							+/-	+/-	+/-
L. plantarum	3692	2MII8					+	+					+	+	+	+					+		+		+	+	+	+	+	+	+	+	+	+	+		+	+				+	+					+/-		+/-		
L. plantarum	3251	2MII11a					+	+	+			+	+	+	+	+					+	+	+	+	+	+	+	+	+	+	+	+	+	+	+			+				+								+/-		
L. plantarum	3252	2MII12a					+	+					+	+	+	+		+/-			+	+	+		+	+	+	+	+	+	+	+	+	+	+		+	+				+/-	+		+					+/-		
L. plantarum	3693	2MIII2					+	+	+/-				+	+	+	+					+		+		+	+	+	+	+	+	+	+	+	+	+		+	+				+	+					+/-		+/-		
L. plantarum	3253	2MIII3						+					+	+	+	+					+	+			+	+	+	+	+	+	+	+	+	+	+			+				+								+/-		
L. plantarum	3254	6PII3B						+					+	+	+	+					+	+	+/-		+	+	+	+	+	+	+	+/-	+	+	+			+				+										
L. plantarum	3694	6PIII1B					+	+					+	+	+	+					+				+	+	+	+	+	+	+	+	+	+	+		+	+					+					+/-		+/-		
L. plantarum	3255	**6PIII6B**						+					+	+	+	+					+	+			+	+	+	+	+	+	+	+	+	+	+			+				+										
L. brevis	3224	2MI2a					+	+	+				+	+	+													+			+		+																			
L. brevis	3225	2MII4											+	+	+	+									+	+		+	+	+	+	+		+								+										
L. brevis	3226	2MII10					+	+	+			+	+	+											+/-			+			+		+																			
L. brevis	3227	**2MIII4**					+	+	+			+	+	+	+											+/-		+			+		+																			
L. brevis	3228	2MIII8					+	+	+			+	+	+	+									+	+/-			+			+		+																			
L. brevis	3229	6PII5					+	+	+				+	+	+									+	+/-			+			+		+																			
L. brevis	3230	6PIII5					+	+	+				+	+	+									+	+/-			+			+		+																			
C. paralimentarius	3239	2MIII5b						+						+	+	+									+	+	+	+	+	+	+			+	+							+										
C. paralimentarius	3240	2MIII6		+/-			+	+	+			+		+	+	+									+	+	+	+	+	+	+			+	+							+								+/-		
C. crustorum	3233	2MI4					+	+					+	+	+	+					+	+	+		+	+	+	+	+	+	+	+	+	+	+		+	+				+	+							+/-		
C. crustorum	3234	2MII6											+	+	+	+									+	+		+	+	+	+	+/-		+			+					+										
C. crustorum	3235	2MII9c					+/-	+/-					+	+	+	+					+	+	+/-	+	+	+/-		+	+	+	+	+	+	+	+		+	+				+	+							+/-		
C. crustorum	3236	2MIII1					+	+					+	+	+	+					+	+	+		+	+	+	+	+	+	+	+	+	+	+		+	+				+/-	+/-							+/-		
C. heilongjiangensis	3237	2MII9b											+	+	+	+								+	+	+		+	+	+	+	+		+	+							+	+/-		+							
C. heilongjiangensis	3238	2MIII5a											+	+	+	+								+	+			+	+	+	+	+			+		+					+										
L. coryniformis	3231	2MII11c											+	+	+	+	+	+		+	+				+		+	+	+		+			+																		

Groups identified based on sequencing of 16S rRNA genes	IBB PAS culture collection no.	Strain	Negative control	Glycerol	Erythritol	D-arabinose	L-arabinose	D-ribose	D-xylose	L-xylose	D-adonitol	β methyl-D-xyloside	D-galactose	D-glucose	D-fructose	D-mannose	L-sorbose	L-rhamnose	Dulcitol	Inozitol	D-mannitol	D-sorbitol	α methyl-D-mannoside	α methyl-D-glucoside	N-acetyl-glucosamine	Amygdalin	Arbutin	Esculin	Salicin	D-cellobiose	D-maltose	D-lactose	D-melibiose	D-sucrose	D-trehalose	Inulin	D-melezitose	D-raffinose	Starch	Glycogen	Xylitol	β-gentiobiose	D-turanose	D-lyxose	D-tagatose	D-fucose	L-fucose	D-arabitol	L-arabitol	Gluconate	2-keto-gluconate	5-keto-gluconate
P. pentosaceus	3267	2MI2c					+	+					+	+	+	+		+/-							+	+	+	+	+	+	+				+							+			+							
P. pentosaceus	3695	2MI7					+	+					+	+	+	+		+			+				+	+	+	+	+	+	+			+	+		+					+			+							
P. pentosaceus	3268	2MI12					+	+	+				+	+	+	+		+			+	+			+	+	+	+	+	+	+	+		+	+		+					+			+							
P. pentosaceus	3269	2MII7					+	+					+	+	+	+		+							+	+	+	+	+	+	+				+							+			+							
P. pentosaceus	3271	2MIII9					+	+					+	+	+	+		+							+	+	+	+	+	+	+				+							+			+							
P. pentosaceus	3696	2MIII10					+	+					+	+	+	+		+							+	+	+	+	+	+	+				+							+			+							
P. pentosaceus	3272	6PII3A					+	+	+				+	+	+	+									+	+	+	+	+	+	+	+			+							+										
P. pentosaceus	3273	6PII4					+	+	+				+	+	+	+									+	+	+	+	+	+	+				+							+										
P. pentosaceus	3274	6PIII1A					+	+	+				+	+	+	+									+	+	+	+	+	+	+		+	+	+			+				+										
P. pentosaceus	3697	6PIII6A					+	+					+	+	+	+		+							+	+	+	+	+	+	+		+		+							+			+							
P. pentosaceus	3275	6PIII7					+	+	+				+	+	+	+									+	+	+	+	+	+	+	+/-			+							+/-			+							
Weissella sp.	3281	2EI1						+/-	+				+	+	+	+									+	+	+	+	+	+	+			+								+/-								+/-		
Weissella sp.	3282	**6PI3**						+/-	+				+	+	+	+									+	+	+	+	+	+	+			+								+/-								+/-	+/-	
Weissella sp.	3283	6PI5A							+				+	+	+	+									+			+		+	+	+	+	+				+				+/-								+/-		
Weissella sp.	3284	6PI5B							+				+	+	+	+									+	+/-	+	+	+/-	+	+			+								+/-								+/-	+/-	
Weissella sp.	3285	6PI6A							+				+	+	+	+									+	+/-		+		+	+											+/-								+/-		
Weissella sp.	3286	6PI6B							+				+	+	+	+									+	+	+/-	+	+/-	+	+											+/-								+/-		
Weissella sp.	3287	6PI7						+/-	+				+	+	+	+									+	+	+	+	+	+	+			+								+/-								+/-	+/-	
Leuconostoc sp.	3263	2EI2						+	+				+	+	+	+									+	+	+	+	+	+	+	+	+	+				+				+/-								+/-		
Leuconostoc sp.	-	2EI3=2EI2						+	+				+	+	+	+									+	+	+	+	+	+	+	+	+	+				+				+/-								+/-		
Leuconostoc sp.	3264	2EII1							+				+	+	+	+									+/-	+/-		+	+/-	+	+		+	+				+				+/-										
L. lactis	3259	2MI6						+	+				+	+	+	+					+				+	+	+	+	+	+	+	+		+	+				+			+										
L. lactis	3261	2BI3						+	+				+	+	+	+					+				+	+	+	+	+	+	+	+		+	+				+/-			+								+/-		
L. lactis	3260	2BI1						+	+				+	+	+	+					+				+	+	+	+	+	+	+	+		+	+				+/-			+/-								+/-		
L. lactis	3698	2BI2						+	+				+	+	+	+					+				+	+	+	+	+	+	+	+		+	+				+			+								+/-		
L. lactis	3262	2BII1						+	+				+	+	+	+					+				+	+	+	+	+	+	+	+		+	+				+/-			+/-										
L. lactis	-	2BII3=2BI3						+	+				+	+	+	+					+				+	+	+	+	+	+	+	+		+	+				+/-			+								+/-		
