# Supplementary material for: Developing lactic acid bacteria starter cultures for wholemeal rye flour bread with improved functionality, nutritional value, taste, appearance and safety
Source: PLoS One. 2022 Jan 14;17(1):e0261677. doi: 10.1371/journal.pone.0261677 (PMC8759695; doi:10.1371/journal.pone.0261677)
Supplement: S1 File — (PDF) [file pone.0261677.s004.pdf]

Chemical composition

Name of starter cultur	technology	code	the moisture content of air-dried bread	Protein [%] x 6,26 in air-dried bread	Ash [%] 550°C in air-dried bread	Dietary fibre - Insoluble fraction in air-dried bread	Dietary fibre – Soluble fraction in air-dried bread	Dietary fibre – Total in air-dried bread	Crumb TTA
**K**	S	VKZ LV2	8,72	7,73	3,37	10,40	4,37	14,77	9
**K**	S	VKZ LV2	8,68	7,72	3,34	10,50	4,31	14,81	9
**K**	D	VKDZ LV2	9,43	7,67	3,31	11,30	3,60	14,90	5
**K**	D	VKDZ LV2	9,44	7,66	3,32	11,26	3,74	15,00	5
**11**	S	VKZ 11	8,25	7,63	3,32	10,69	4,64	15,33	8
**11**	S	VKZ 11	8,28	7,83	3,35	10,57	4,69	15,26	8
**11**	D	VKDZ 11	9,10	7,69	3,31	11,24	3,86	15,09	5
**11**	D	VKDZ 11	9,04	7,63	3,33	11,18	3,76	14,94	5
**12**	S	VKZ 12	9,18	7,85	3,37	10,40	4,01	14,41	10
**12**	S	VKZ 12	9,20	7,79	3,36	10,46	4,12	14,58	10
**12**	D	VKDZ 12	10,14	7,68	3,38	10,88	3,93	14,81	6
**12**	D	VKDZ 12	10,10	7,65	3,37	10,88	4,02	14,90	6
**12b**	S	VKZ 12B	7,88	9,14	3,50	10,19	4,28	14,47	8
**12b**	S	VKZ 12B	7,82	9,18	3,53	10,11	4,25	14,36	8
**12b**	D	VKDZ 12B	8,00	9,40	3,52	10,98	4,49	15,47	5
**12b**	D	VKDZ 12B	7,97	9,33	3,50	10,94	4,45	15,39	5

Organic acids

Sample	CODE	Name	technology	rep	Sample Amount	TART [%]	CITR	MALIC	LAC	ACE	PROP	SUM OA [%]	Sum LA	%Lac
**VKZ_LV2 x1**	VKZ_LV2	K	S	1	471,7	0,0081	0,0057	0,0333	0,3735	0,1605	0,1193	0,700	0,53	69,9
**VKZ_LV2 x2**	VKZ_LV2	K	S	2	471,7	0,0081	0,0056	0,0173	0,3876	0,1616	0,125	0,705	0,55	70,6
**VKZ_11 x1**	VKZ_11	11	S	1	471,7	0,0045	0,0003	0,0428	0,5562	0,0798	0,1577	0,841	0,64	87,5
**VKZ_11 x2**	VKZ_11	11	S	2	471,7	0,0084	0,0005	0,043	0,5401	0,0752	0,1507	0,818	0,62	87,8
**VKZ_12 x2**	VKZ_12	12	S	1	471,7	0,0089	0,0015	0,0039	0,422	0,1522	0,066	0,655	0,57	73,5
**VKZ_12 x1**	VKZ_12	12	S	2	471,7	0,0081	0,0017	0,0104	0,4376	0,1403	0,0838	0,682	0,58	75,7
**VKZ12B x2**	VKZ_12B	12B	S	1	471,7	0,0074	0,0015	0,0224	0,4523	0,0224	0,079	0,585	0,47	95,3
**VKZ12B x1**	VKZ_12B	12B	S	2	471,7	0,0073	0,0014	0,0266	0,48	0,0187	0,0845	0,619	0,50	96,3
**VKDZ_LV2 x1**	VKDZ_LV2	K	D	1	471,7	0,0091	0,0094	0,0262	0,2349	0,1126	0,1222	0,514	0,35	67,6
**VKDZ_LV2 x2**	VKDZ_LV2	K	D	2	471,7	0,008	0,0078	0,0192	0,2477	0,1092	0,145	0,537	0,36	69,4
**VKDZ_11 x1**	VKDZ_11	11	D	1	471,7	0	0,0026	0,042	0,3602	0,0565	0,1582	0,620	0,42	86,4
**VKDZ_11 x2**	VKDZ_11	11	D	2	471,7	0	0,0027	0,0524	0,3495	0,0583	0,1506	0,614	0,41	85,7
**VKDZ_12 x2**	VKDZ_12	12	D	1	471,7	0,0076	0,0033	0,0254	0,2409	0,0757	0,0838	0,437	0,32	76,1
**VKDZ_12 x1**	VKDZ_12	12	D	2	471,7	0,01	0,004	0,0172	0,2441	0,0769	0,0683	0,421	0,32	76,0
**VKDZ_12B x2**	VKDZ_12B	12B	D	1	471,7	0	0,0037	0,0199	0,2663	0,0269	0,0704	0,387	0,29	90,8
**VKDZ_12B x1**	VKDZ_12B	12B	D	2	471,7	0	0,0039	0,0246	0,2565	0,0243	0,0693	0,379	0,28	91,3

Phytate

CODE	Name	technology	rep	IP6 (%w.b)	Sum IP (%w.b)	d.b. M [%]	sample M	IP6 (% d.b.M)	Sum IP (%d.b.M)	d.b.	sample weight	IP6 (% d.b.)	Sum IP (%d.b)
**VKZ_LV2**	K	S	1	0,05	0,25	56,99	569,90	0,10	0,44	56,49	564,90	0,10	0,44
**VKZ_LV2**	K	S	2	0,06	0,25	56,99	569,90	0,10	0,44	57,39	573,90	0,10	0,43
**VKZ_11**	11	S	1	0,04	0,17	61,27	673,97	0,07	0,28	60,27	662,97	0,07	0,29
**VKZ_11**	11	S	2	0,04	0,18	61,27	673,97	0,07	0,29	62,27	684,97	0,06	0,28
**VKZ_12**	12	S	1	0,01	0,13	53,05	583,55	0,03	0,25	50,05	550,55	0,03	0,27
**VKZ_12**	12	S	2	0,01	0,13	53,05	583,55	0,03	0,25	56,05	616,55	0,03	0,24
**VKZ_12B**	12B	S	1	0,15	0,42	51,55	567,05	0,29	0,82	49,55	545,05	0,30	0,85
**VKZ_12B**	12B	S	2	0,14	0,40	51,55	567,05	0,27	0,77	53,55	589,05	0,26	0,74
**VKDZ_LV2**	K	D	1	0,19	0,39	55,76	557,60	0,35	0,69	55,36	553,60	0,35	0,70
**VKDZ_LV2**	K	D	2	0,22	0,39	55,76	557,60	0,39	0,69	56,16	561,60	0,38	0,69
**VKDZ_11**	11	D	1	0,22	0,41	61,67	678,37	0,35	0,66	58,67	645,37	0,37	0,69
**VKDZ_11**	11	D	2	0,19	0,37	61,67	678,37	0,31	0,61	64,67	711,37	0,29	0,58
**VKDZ_12**	12	D	1	0,11	0,26	53,54	588,94	0,20	0,49	55,54	610,94	0,19	0,48
**VKDZ_12**	12	D	2	0,11	0,27	53,54	588,94	0,20	0,50	51,54	566,94	0,21	0,52
**VKDZ_12B**	12B	D	1	0,26	0,37	57,35	630,85	0,45	0,64	57,00	627,00	0,45	0,65
**VKDZ_12B**	12B	D	2	0,25	0,37	57,35	630,85	0,44	0,64	57,70	634,70	0,44	0,64

Alcohols and sugars

Code	Name	technology	rep	Mal [%]	Glc [%]	Glyc [%]	EtOH [%]
**VKZ_LV2**	K	S	1	0,5569	0,4411	0,1119	0,1971
**VKZ_LV2**	K	S	2	0,54	0,4301	0,1089	0,2255
**VKZ_11**	11	S	1	0,8676	0,6336	0,1595	0,1992
**VKZ_11**	11	S	2	0,8802	0,6352	0,1414	0,2019
**VKZ_12**	12	S	1	0,4134	0,3044	0,1584	0,2307
**VKZ_12**	12	S	2	0,4298	0,3184	0,1444	0,2372
**VKZ_12B**	12B	S	1	0,8665	0,4464	0,1314	0,244
**VKZ_12B**	12B	S	2	0,8979	0,4725	0,1418	0,2522
**VKDZ_LV2**	K	D	1	0,6272	0,3771	0,1782	0,2381
**VKDZ_LV2**	K	D	2	0,6228	0,3826	0,1852	0,2376
**VKDZ_11**	11	D	1	0,5562	0,3994	0,1996	0,2687
**VKDZ_11**	11	D	2	0,5596	0,405	0,19	0,2613
**VKDZ_12**	12	D	1	0,3531	0,2343	0,1127	0,2357
**VKDZ_12**	12	D	2	0,3389	0,2205	0,1127	0,2357
**VKDZ_12**	12	D	3	0,3704	0,245	0,1184	0,2372
**VKDZ_12B**	12B	D	1	0,5834	0,2526	0,159	0,2745
**VKDZ_12B**	12B	D	2	0,5567	0,2351	0,1371	0,2663

Quality

CODE	Name	technology	Weight g]	Volume [cm^3]	Volume of 100 g of bread [cm^3]	dough yield	Baking loss [%]	Bread yield
**VKZ LV2**	K	S	521,44	773,799	148,39656	174,13	13,0933	151,331
**VKZ LV2**	K	S	527,81	801,875	151,92494	174,13	12,0317	153,179
**VKZ LV2**	K	S	520,09	777,798	149,55065	174,13	13,3183	150,939
**VKZ LV2**	K	S	519,06	791,290	152,44673	174,13	13,49	150,64
**VKZ LV2**	K	S	527,68	767,940	145,53138	174,13	12,0533	153,142
**VKZ LV2**	K	S	516,63	786,592	152,25442	174,13	13,895	149,935
**VKZ LV2**	K	S	529,37	766,713	144,83499	174,13	11,7717	153,632
**VKZ LV2**	K	S	527,02	796,272	151,08952	174,13	12,1633	152,95
**VKZ LV2**	K	S	519,75	764,913	147,16941	174,13	13,375	150,84
**VKDZ LV2**	K	D	518,95	831,746	160,27479	175,09	13,5083	151,438
**VKDZ LV2**	K	D	524,26	857,231	163,51257	175,09	12,6233	152,988
**VKDZ LV2**	K	D	514,62	849,057	164,98718	175,09	14,23	150,175
**VKDZ LV2**	K	D	510,62	865,319	169,46438	175,09	14,8967	149,007
**VKDZ LV2**	K	D	514,66	841,198	163,44732	175,09	14,2233	150,186
**VKDZ LV2**	K	D	511,88	831,428	162,42635	175,09	14,6867	149,375
**VKDZ LV2**	K	D	515,14	853,871	165,75513	175,09	14,1433	150,326
**VKDZ LV2**	K	D	505,79	848,651	167,78722	175,09	15,7017	147,598
**VKDZ LV2**	K	D	509,16	863,189	169,53197	175,09	15,14	148,581
**VKZ 11**	11	S	517,86	824,989	159,30734	174,13	13,69	150,292
**VKZ 11**	11	S	531,88	816,661	153,54234	174,13	11,3533	154,36
**VKZ 11**	11	S	526,51	812,240	154,26867	174,13	12,2483	152,802
**VKZ 11**	11	S	517,32	822,264	158,94688	174,13	13,78	150,135
**VKZ 11**	11	S	518,91	803,286	154,80257	174,13	13,515	150,596
**VKZ 11**	11	S	528,77	804,990	152,23821	174,13	11,8717	153,458
**VKZ 11**	11	S	521,62	798,025	152,98972	174,13	13,0633	151,383
**VKZ 11**	11	S	518,68	829,125	159,8529	174,13	13,5533	150,53

**VKZ 11**	11	S	523,53	825,860	157,74836	174,13	12,745	151,937
**VKDZ 11**	11	D	516,86	829,255	160,44093	175,09	13,8567	150,828
**VKDZ 11**	11	D	520,38	863,964	166,0256	175,09	13,27	151,856
**VKDZ 11**	11	D	506,27	837,060	165,33865	175,09	15,6217	147,738
**VKDZ 11**	11	D	516,52	858,433	166,1955	175,09	13,9133	150,729
**VKDZ 11**	11	D	520,96	858,853	164,85968	175,09	13,1733	152,025
**VKDZ 11**	11	D	511,39	831,384	162,57338	175,09	14,7683	149,232
**VKDZ 11**	11	D	525,58	834,194	158,71875	175,09	12,4033	153,373
**VKDZ 11**	11	D	522,56	873,504	167,1586	175,09	12,9067	152,492
**VKDZ 11**	11	D	508,64	835,015	164,16621	175,09	15,2267	148,43
**VKZ 12**	12	S	516,34	808,763	156,63381	174,13	13,9433	149,85
**VKZ 12**	12	S	518,70	824,956	159,04299	174,13	13,55	150,535
**VKZ 12**	12	S	513,39	818,954	159,51888	174,13	14,435	148,994
**VKZ 12**	12	S	513,16	792,514	154,43799	174,13	14,4733	148,928
**VKZ 12**	12	S	512,79	805,501	157,08204	174,13	14,535	148,82
**VKZ 12**	12	S	520,05	829,863	159,57369	174,13	13,325	150,927
**VKZ 12**	12	S	516,70	829,811	160,59822	174,13	13,8833	149,955
**VKZ 12**	12	S	524,03	830,736	158,52833	174,13	12,6617	152,082
**VKZ 12**	12	S	524,51	815,174	155,41629	174,13	12,5817	152,222
**VKDZ 12**	12	D	513,37	867,315	168,9454	175,09	14,4383	149,81
**VKDZ 12**	12	D	520,07	831,669	159,91482	175,09	13,3217	151,765
**VKDZ 12**	12	D	514,33	854,884	166,21313	175,09	14,2783	150,09
**VKDZ 12**	12	D	523,05	851,003	162,70012	175,09	12,825	152,635
**VKDZ 12**	12	D	505,25	852,459	168,72024	175,09	15,7917	147,44
**VKDZ 12**	12	D	507,09	845,371	166,71025	175,09	15,485	147,977
**VKDZ 12**	12	D	512,99	862,329	168,0986	175,09	14,5017	149,699
**VKDZ 12**	12	D	510,42	845,845	165,71549	175,09	14,93	148,949
**VKDZ 12**	12	D	515,07	862,163	167,38754	175,09	14,155	150,306
**VKZ 12B**	12B	S	511,08	810,845	158,65324	174,13	14,82	148,324
**VKZ 12B**	12B	S	506,57	809,108	159,72284	174,13	15,5717	147,015

**VKZ 12B**	12B	S	510,20	822,863	161,28244	174,13	14,9667	148,069
**VKZ 12B**	12B	S	512,45	801,657	156,43614	174,13	14,5917	148,722
**VKZ 12B**	12B	S	509,20	818,454	160,73331	174,13	15,1333	147,778
**VKZ 12B**	12B	S	495,57	804,351	162,30825	174,13	17,405	143,823
**VKZ 12B**	12B	S	504,35	822,660	163,11292	174,13	15,9417	146,371
**VKZ 12B**	12B	S	509,82	807,171	158,3247	174,13	15,03	147,958
**VKZ 12B**	12B	S	514,14	824,998	160,46174	174,13	14,31	149,212
**VKDZ 12B**	12B	D	505,22	889,725	176,10645	175,09	15,7967	147,432
**VKDZ 12B**	12B	D	506,30	877,025	173,2224	175,09	15,6167	147,747
**VKDZ 12B**	12B	D	501,15	895,195	178,62816	175,09	16,475	146,244
**VKDZ 12B**	12B	D	508,89	886,597	174,22174	175,09	15,185	148,503
**VKDZ 12B**	12B	D	520,92	906,364	173,99294	175,09	13,18	152,013
**VKDZ 12B**	12B	D	511,70	889,152	173,76432	175,09	14,7167	149,323
**VKDZ 12B**	12B	D	496,80	872,535	175,63104	175,09	17,2	144,975
**VKDZ 12B**	12B	D	497,64	877,856	176,40383	175,09	17,06	145,22
**VKDZ 12B**	12B	D	501,07	882,795	176,18197	175,09	16,4883	146,221

Texture and moisture

CODE	Name	technology	day of storage	moisture of the crumb	Hardness	Springiness	Cohesiveness	Chewiness	Resilience
**VKZ LV2 a**	K	S	0	46,37	53,2401	0,93911	0,3825	19,1243	0,10979
**VKZ LV2 a**	K	S	0	46,39	53,7668	0,99585	0,36272	19,4212	0,1184
**VKZ LV2 b**	K	S	1	46,41	50,8202	0,92949	0,30333	14,3285	0,08863
**VKZ LV2 b**	K	S	1	46,47	47,8687	0,93016	0,29246	13,0222	0,07842
**VKZ LV2 c**	K	S	4	46,89	70,6424	0,93579	0,30948	20,4585	0,08933
**VKZ LV2 c**	K	S	4	46,74	71,6596	0,97297	0,29998	20,9156	0,08658
**VKZ LV2 d**	K	S	5	45,96	71,6687	0,894	0,30635	19,6285	0,08648
**VKZ LV2 d**	K	S	5	45,76	73,2584	0,954	0,30209	21,1124	0,08612
**VKDZ LV2 a**	K	D	0	49,75	37,1427	0,90196	0,33251	11,1396	0,0957
**VKDZ LV2 a**	K	D	0	49,76	39,4753	0,97383	0,31869	12,251	0,10118
**VKDZ LV2 b**	K	D	1	49,60	33,6135	0,83399	0,29187	8,18209	0,09009
**VKDZ LV2 b**	K	D	1	49,51	31,6254	0,93659	0,26899	7,96756	0,08259
**VKDZ LV2 c**	K	D	4	48,83	42,8485	0,88784	0,25627	9,74907	0,07398
**VKDZ LV2 c**	K	D	4	48,94	39,8914	0,89739	0,25125	8,99432	0,07116
**VKDZ LV2 d**	K	D	5	48,02	44,5452	0,95027	0,28101	11,8952	0,07623
**VKDZ LV2 d**	K	D	5	47,90	38,8428	0,89754	0,25474	8,88109	0,07412
**VKZ 11 a**	11	S	0	47,55	39,5501	0,94454	0,37279	13,9263	0,11509
**VKZ 11 a**	11	S	0	47,58	40,2823	0,90245	0,35778	13,0065	0,11106
**VKZ 11 b**	11	S	1	47,22	39,759	0,94405	0,31039	11,6504	0,08081
**VKZ 11 b**	11	S	1	47,21	41,9794	0,90656	0,31907	12,1428	0,08588
**VKZ 11 c**	11	S	4	47,24	45,7523	0,94952	0,2756	11,9729	0,08635
**VKZ 11 c**	11	S	4	47,12	45,2012	0,90284	0,28449	11,61	0,07445
**VKZ 11 d**	11	S	5	46,78	64,2118	0,98368	0,33614	21,2323	0,08425
**VKZ 11 d**	11	S	5	46,91	56,4022	0,98062	0,29692	16,4224	0,08009
**VKDZ 11 a**	11	D	0	47,32	43,9539	0,95191	0,40002	16,7367	0,11136
**VKDZ 11 a**	11	D	0	47,28	42,1694	0,99354	0,36258	15,191	0,10488
**VKDZ 11 b**	11	D	1	48,88	36,8797	0,96929	0,28166	10,0686	0,07884

**VKDZ 11 b**	11	D	1	48,73	37,1299	0,92483	0,29223	10,0348	0,08551
**VKDZ 11 c**	11	D	4	48,7	44,5733	0,8882	0,29668	11,7457	0,08218
**VKDZ 11 c**	11	D	4	48,65	44,5684	0,91164	0,26618	10,8151	0,08467
**VKDZ 11 d**	11	D	5	48,37	53,252	0,79887	0,34292	14,5882	0,09538
**VKDZ 11 d**	11	D	5	48,24	52,5219	0,94973	0,29122	14,5268	0,08715
**VKZ 12 a**	12	S	0	48,12	32,1276	0,96491	0,37164	11,5209	0,11211
**VKZ 12 a**	12	S	0	48,03	32,6646	0,98273	0,36135	11,5996	0,10655
**VKZ 12 b**	12	S	1	47,80	33,8555	0,87523	0,31993	9,48003	0,09912
**VKZ 12 b**	12	S	1	47,70	34,2666	0,92144	0,31339	9,89535	0,09293
**VKZ 12 c**	12	S	4	47,56	50,5115	0,93103	0,30898	14,5307	0,08131
**VKZ 12 c**	12	S	4	47,73	49,2067	0,96793	0,32882	15,661	0,08115
**VKZ 12 d**	12	S	5	48,07	47,6165	0,93367	0,29976	13,3267	0,07932
**VKZ 12 d**	12	S	5	48,22	43,3328	0,98747	0,29329	12,5497	0,07781
**VKDZ 12 a**	12	D	0	50,16	28,913	0,99419	0,36897	10,606	0,11102
**VKDZ 12 a**	12	D	0	50,26	30,4255	0,99645	0,37421	11,3451	0,11407
**VKDZ 12 b**	12	D	1	49,72	30,7542	0,88282	0,31162	8,46068	0,09622
**VKDZ 12 b**	12	D	1	49,70	27,4711	0,89614	0,28746	7,07659	0,08943
**VKDZ 12 c**	12	D	4	49,73	42,802	0,90863	0,28867	11,2268	0,08573
**VKDZ 12 c**	12	D	4	49,63	40,2203	0,96688	0,3315	12,8915	0,08945
**VKDZ 12 d**	12	D	5	49,20	35,7295	1,00174	0,31839	11,3959	0,07311
**VKDZ 12 d**	12	D	5	49,29	37,2889	0,95139	0,27816	9,86811	0,07297
**VKZ 12B a**	12B	S	0	49,72	23,128	0,961	0,347	7,702	0,097
**VKZ 12B a**	12B	S	0	49,74	26,492	0,922	0,415	10,13	0,112
**VKZ 12B b**	12B	S	1	49,67	25,629	0,904	0,326	7,56	0,09
**VKZ 12B b**	12B	S	1	49,84	24,709	0,997	0,365	8,999	0,092
**VKZ 12B c**	12B	S	4	49,31	34,495	0,867	0,315	9,429	0,079
**VKZ 12B c**	12B	S	4	49,33	35,411	0,931	0,318	10,491	0,08
**VKZ 12B d**	12B	S	5	49,34	37,679	0,989	0,366	13,63	0,083
**VKZ 12B d**	12B	S	5	49,16	34,516	1	0,305	10,511	0,072
**VKDZ 12B a**	12B	D	0	51,13	21,104	0,994	0,377	7,9	0,093

**VKDZ 12B a**	12B	D	0	51,07	23,59	0,984	0,392	9,098	0,099
**VKDZ 12B b**	12B	D	1	50,90	21,895	0,966	0,313	6,61	0,07
**VKDZ 12B b**	12B	D	1	50,64	21,343	0,918	0,313	6,127	0,074
**VKDZ 12B c**	12B	D	4	50,57	29,006	0,963	0,282	7,874	0,069
**VKDZ 12B c**	12B	D	4	50,8	27,24	0,959	0,282	7,371	0,07
**VKDZ 12B d**	12B	D	5	50,81	29,682	0,954	0,259	7,338	0,062
**VKDZ 12B d**	12B	D	5	50,74	30,149	0,931	0,278	7,817	0,065

Organoleptic assesment

Code			appearance	color of the crust	thickness of the crust	other characteristics of the skin	elasticity of the crumb	porosity of the crumb	color and cutting ability of the crumb	teast	sum of poit	sum with point for quality (8 pkt)
**VKZ LV2**	K	S	4	2	3	3	3	2	2	5	24	32
**VKZ LV2**	K	S	5	3	4	4	3	3	3	5	30	38
**VKZ LV2**	K	S	4	3	3	3	3	3	2	5	26	34
**VKZ LV2**	K	S	5	3	3	4	3	3	3	5	29	37
**VKZ LV2**	K	S	4	3	3	4	3	2	2	5	26	34
**VKZ LV2**	K	S	4	2	3	4	3	3	2	6	27	35
**VKZ LV2**	K	S	4	2	4	4	3	2	3	6	28	36
**VKZ LV2**	K	S	4	2	4	4	3	2	3	6	28	36
**VKZ LV2**	K	S	4	3	4	4	3	2	3	6	29	37
**VKZ LV2**	K	S	5	2	4	4	4	3	3	6	31	39
**VKZ LV2**	K	S	4	3	4	4	3	3	2	6	29	37
**VKZ LV2**	K	S	5	3	3	3	3	2	3	6	28	36
**VKDZ LV2**	K	D	5	3	4	4	4	2	3	5	30	38
**VKDZ LV2**	K	D	5	3	3	3	4	2	3	5	28	36
**VKDZ LV2**	K	D	4	3	3	3	3	2	2	5	25	33
**VKDZ LV2**	K	D	4	3	3	4	3	2	2	5	26	34
**VKDZ LV2**	K	D	4	3	3	3	3	2	0	5	23	31
**VKDZ LV2**	K	D	4	2	3	3	3	0	2	5	22	30
**VKDZ LV2**	K	D	5	3	4	4	3	3	3	6	31	39
**VKDZ LV2**	K	D	5	3	4	4	4	3	3	5	31	39
**VKDZ**	K	D	5	3	4	4	4	3	3	5	31	39

**LV2**												
**VKDZ LV2**	K	D	4	3	3	4	3	2	3	6	28	36
**VKDZ LV2**	K	D	5	3	4	4	4	3	3	5	31	39
**VKZ 11**	11	S	5	2	3	3	3	2	3	5	26	34
**VKZ 11**	11	S	5	3	3	3	4	3	3	5	29	37
**VKZ 11**	11	S	5	3	4	4	4	3	3	5	31	39
**VKZ 11**	11	S	5	2	4	4	4	3	3	6	31	39
**VKZ 11**	11	S	5	2	3	3	3	2	2	5	25	33
**VKZ 11**	11	S	5	3	3	3	4	3	3	5	29	37
**VKZ 11**	11	S	5	2	4	4	3	2	3	6	29	37
**VKZ 11**	11	S	5	3	3	4	3	3	3	6	30	38
**VKZ 11**	11	S	5	2	3	4	4	3	2	6	29	37
**VKZ 11**	11	S	5	3	4	4	4	2	2	5	29	37
**VKZ 11**	11	S	5	3	3	4	3	0	2	5	25	33
**VKZ 11**	11	S	5	3	3	3	4	2	2	5	27	35
**VKDZ 11**	11	D	5	2	3	3	3	2	3	5	26	34
**VKDZ 11**	11	D	5	3	4	4	4	3	3	5	31	39
**VKDZ 11**	11	D	5	3	4	4	4	3	2	5	30	38
**VKDZ 11**	11	D	5	2	4	4	4	3	3	5	30	38
**VKDZ 11**	11	D	5	2	3	3	3	3	2	5	26	34
**VKDZ 11**	11	D	5	3	4	4	3	3	3	5	30	38
**VKDZ 11**	11	D	5	2	3	3	3	2	2	5	25	33
**VKDZ 11**	11	D	5	3	3	4	3	3	2	6	29	37
**VKDZ 11**	11	D	5	3	4	4	3	2	2	6	29	37
**VKDZ 11**	11	D	4	2	3	4	3	2	2	5	25	33
**VKDZ 11**	11	D	5	2	3	3	3	3	2	6	27	35

**VKZ 12**	12	S	5	3	4	4	3	3	3	5	30	38
**VKZ 12**	12	S	5	2	4	4	3	2	3	5	28	36
**VKZ 12**	12	S	5	2	4	3	3	2	2	5	26	34
**VKZ 12**	12	S	5	3	4	4	3	2	2	5	28	36
**VKZ 12**	12	S	5	2	3	4	4	2	2	5	27	35
**VKZ 12**	12	S	5	3	4	4	0	2	2	5	25	33
**VKZ 12**	12	S	5	3	4	4	3	2	3	5	29	37
**VKZ 12**	12	S	5	2	4	4	3	3	2	5	28	36
**VKZ 12**	12	S	5	3	4	4	4	2	2	5	29	37
**VKZ 12**	12	S	5	2	4	4	3	3	2	5	28	36
**VKZ 12**	12	S	5	3	3	4	3	2	3	0	23	31
**VKDZ 12**	12	D	4	3	4	4	3	2	2	5	27	35
**VKDZ 12**	12	D	5	3	4	4	3	3	3	6	31	39
**VKDZ 12**	12	D	5	2	4	4	4	3	3	6	31	39
**VKDZ 12**	12	D	5	3	4	4	3	3	3	6	31	39
**VKDZ 12**	12	D	5	3	4	4	3	3	3	6	31	39
**VKDZ 12**	12	D	5	3	4	4	4	2	3	5	30	38
**VKDZ 12**	12	D	5	3	4	4	4	2	3	5	30	38
**VKDZ 12**	12	D	5	3	4	4	3	3	2	6	30	38
**VKDZ 12**	12	D	5	3	4	4	4	2	3	5	30	38
**VKDZ 12**	12	D	5	3	4	4	3	2	2	6	29	37
**VKDZ 12**	12	D	5	3	4	4	4	3	3	6	32	40
**VKDZ 12**	12	D	5	3	4	4	4	2	3	6	31	39
**VKDZ 12**	12	D	5	3	4	4	4	2	2	6	30	38
**VKDZ 12**	12	D	5	3	4	4	4	3	3	6	32	40

**VKDZ 12**	12	D	5	3	4	4	4	2	2	6	30	38
**VKZ 12B**	12 B	S	4	2	4	4	4	3	3	6	30	38
**VKZ 12B**	12 B	S	4	2	4	4	4	3	3	6	30	38
**VKZ 12B**	12 B	S	5	3	4	4	4	3	3	5	31	39
**VKZ 12B**	12 B	S	5	2	4	4	3	2	2	6	28	36
**VKZ 12B**	12 B	S	5	3	3	3	4	3	2	6	29	37
**VKZ 12B**	12 B	S	5	3	3	4	4	2	3	6	30	38
**VKZ 12B**	12 B	S	4	2	4	4	4	3	3	5	29	37
**VKZ 12B**	12 B	S	5	3	4	4	4	3	3	5	31	39
**VKZ 12B**	12 B	S	5	2	4	4	3	2	2	6	28	36
**VKZ 12B**	12 B	S	5	3	3	3	4	3	2	6	29	37
**VKZ 12B**	12 B	S	5	3	3	4	4	2	3	6	30	38
**VKZ 12B**	12 B	S	4	2	4	4	4	3	3	5	29	37
**VKZ 12B**	12 B	S	4	2	4	4	4	3	3	6	30	38
**VKZ 12B**	12 B	S	4	2	4	4	4	3	3	6	30	38
**VKDZ 12B**	12 B	D	4	3	4	4	3	3	3	5	29	37
**VKDZ 12B**	12 B	D	5	3	4	4	3	3	3	6	31	39
**VKDZ 12B**	12 B	D	4	3	4	4	4	2	3	5	29	37
**VKDZ 12B**	12 B	D	5	3	4	3	4	2	3	6	30	38
**VKDZ 12B**	12 B	D	4	3	4	4	4	3	3	5	30	38
**VKDZ 12B**	12 B	D	4	3	4	4	4	3	3	5	30	38
**VKDZ 12B**	12 B	D	5	3	4	3	4	2	3	6	30	38
**VKDZ 12B**	12 B	D	4	3	4	4	4	3	3	5	30	38

**VKDZ 12B**	12B	D	5	3	4	4	3	3	3	6	31	39
**VKDZ 12B**	12B	D	4	3	4	4	3	3	3	5	29	37
**VKDZ 12B**	12B	D	4	3	4	4	4	2	3	5	29	37
**VKDZ 12B**	12B	D	4	3	4	4	4	3	3	5	30	38
**VKDZ 12B**	12B	D	4	3	4	4	4	3	3	5	30	38
